# The extracellular matrix molecule tenascin-C modulates cell cycle progression and motility of adult neural stem/progenitor cells from the subependymal zone

**DOI:** 10.1007/s00018-022-04259-5

**Published:** 2022-04-16

**Authors:** Elena Schaberg, Magdalena Götz, Andreas Faissner

**Affiliations:** 1 Department of Cell Morphology and Molecular Neurobiology, Ruhr-University Bochum, Universitätsstrasse 150, 44780 Bochum, Germany; 2 Physiological Genomics, Biomedical Center, LMU, Planegg-Martinsried, Germany; 3 Institute of Stem Cell Research, Helmholtz Center Munich, Biomedical Center, LMU, Planegg-Martinsried, Germany; 4 Synergy, Excellence Cluster for Systems Neurology, BMC, LMU, Planegg-Martinsried, Germany

**Keywords:** Adult neurogenesis, Epidermal growth factor receptor (EGFR), Laminin-1, Lineage tracking, Neural lineage, Stem cell niche, Tenascin gene family, Time-lapse video microscopy

## Abstract

Adult neurogenesis has been described in two canonical regions of the adult central nervous system (CNS) of rodents, the subgranular zone (SGZ) of the hippocampus and the subependymal zone (SEZ) of the lateral ventricles. The stem cell niche of the SEZ provides a privileged environment composed of a specialized extracellular matrix (ECM) that comprises the glycoproteins tenascin-C (Tnc) and laminin-1 (LN1). In the present study, we investigated the function of these ECM glycoproteins in the adult stem cell niche. Adult neural stem/progenitor cells (aNSPCs) of the SEZ were prepared from wild type (*Tnc*^+*/*+^) and Tnc knockout (*Tnc*^*−/−*^) mice and analyzed using molecular and cell biological approaches. A delayed maturation of aNSPCs in *Tnc*^*−/−*^ tissue was reflected by a reduced capacity to form neurospheres in response to epidermal growth factor (EGF). To examine a potential influence of the ECM on cell proliferation, aNSPCs of both genotypes were studied by cell tracking using digital video microscopy. aNSPCs were cultivated on three different substrates, namely, poly-d-lysine (PDL) and PDL replenished with either LN1 or Tnc for up to 6 days in vitro. On each of the three substrates aNSPCs displayed lineage trees that could be investigated with regard to cell cycle length. The latter appeared reduced in *Tnc*^*−/−*^ aNSPCs on PDL and LN1 substrates, less so on Tnc that seemed to compensate the absence of the ECM compound to some extent. Close inspection of the lineage trees revealed a subpopulation of late dividing aNSPCs_late_ that engaged into cycling after a notable delay. aNSPCs_late_ exhibited a clearly different morphology, with a larger cell body and conspicuous processes. aNSPCs_late_ reiterated the reduction in cell cycle length on all substrates tested, which was not rescued on Tnc substrates. When the migratory activity of aNSPC-derived progeny was determined, *Tnc*^*−/−*^ neuroblasts displayed significantly longer migration tracks. This was traced to an increased rate of migration episodes compared to the wild-type cells that rested for longer time periods. We conclude that Tnc intervenes in the proliferation of aNSPCs and modulates the motility of neuroblasts in the niche of the SEZ.

**Supplementary Information:**

The online version contains supplementary material available at 10.1007/s00018-022-04259-5.

## Introduction

The adult mammalian brain retains neural stem cells in closely defined areas, the so-called stem cell niches at the lateral ventricle and the subgranular zone of the dentate gyrus [1, 2]. The stem cells from the subependymal zone (SEZ) of the lateral ventricle are surrounded by niche astrocytes, blood vessels and their own generated progeny underneath a dense layer of ependymal cells facing the lumen of the ventricle [3–5]. These stem cells have astroglial characteristics and are also called SVZ astrocytes or type B cells [5–7]. They give rise to type C cells, which are fast-cycling transient amplifying progenitors (TAPs) and in the end produce type A cells which are neuroblasts generated by a final symmetric division [8, 9]. These newborn neurons are integrated into the neuronal network of the olfactory bulb, which they reach by migration through the rostral migratory stream [10]. Recent investigations based on single cell transcriptomics have revealed a considerable heterogeneity of the subependymal zone. This niche comprises stem cells, but also progenitor populations of different lineages, endothelia and support cells totaling up to 17 distinct cell clusters that can be distinguished by bioinformatic analysis of transcriptomes [11–13]. Therefore, we will refer to these cells as adult neural stem/progenitor cells (aNSPCs) for the purpose of this study.

At the beginning, adult neural stem cells were investigated in vitro in neurosphere cultures [14] or in adherent cultures by addition of growth factors or astroglial feeder layers [15, 16]. Subsequent studies could show that the addition of such factors considerably influences the proliferation mode of aNSPCs [9, 17]. On these bases, protocols were developed that allow to study the behavior of aNSPCs from the SEZ in the absence of mitogenic or cell fate-changing factors [8]. This approach unraveled the intrinsic program of lineage progression and opened that possibility to investigate extracellular matrix (ECM) effects by presenting defined niche molecules in vitro [8, 9].

The ECM has been shown to be of great importance for the behavior of diverse cell types from different tissues [18–20]. The glycoprotein tenascin-C (Tnc) is part of the ECM in stem cell niches of the central nervous system in development and adulthood [21–26]. The close association of Tnc with stem cell environments prompted the analysis of Tnc impact on stem cell behavior during development [27–30]. The use of Tnc-knockout cells [29] as well as the cultivation on purified Tnc substrates [31] or the Tnc-derived peptide VSWRAPTA [32] could confirm the hypothesis that Tnc does strongly influence processes, such as cell cycle progression, differentiation and neurite growth in specific contexts of early development.

Another important member of the ECM is laminins as part of the basal lamina. They are expressed in the subventricular zone of adult mice [33] and a promoting influence of laminins on cell behavior has already been reported for myoblasts [34] or neural progenitor cells [35, 36] in terms of proliferation and neurogenesis. We present here a study that investigates the influence of the two extracellular matrix compounds Tnc and laminin-1 on the lineage progression of aNSPCs from the SEZ by time-lapse video microscopy and cell tracking to obtain deeper insights into the direct influence of the niche environment on adult stem cell behavior under specifically defined conditions.

## Materials and methods

### Legal issues and animal housing

The present study was carried out in accordance with the European Council Directive of September 22, 2010 (2010/63/EU) for care of laboratory animals and approved by the animal care committee of North Rhine-Westphalia, Germany, based at the LANUV (Landesamt für Natur, Umwelt und Verbraucherschutz, Nordrhein-Westfalen, D-45659 Recklinghausen, Germany). The study was supervised by the animal welfare commissioner of Ruhr-University. All efforts were made to reduce the number of animals in the experiments. Embryos of both sexes were used. Data of this publication are based on experiments performed with Mus musculus with tenascin-C deficiency. The systemic knockout has initially been generated and published by Forsberg and colleagues [37]. The *Tnc*^*−/−*^ knockout line was compared to wild types from the 129sv mouse strain that shares the same genetic background. Handling of the animals was conducted according to German animal protection laws and Federation for Laboratory Animal Science Associations (FELASA) standards. Mice were kept under controlled conditions with a 12-h light/dark cycle and had access to water and food ad libitum. Adult animals at the age of 9–11 weeks were anesthetized with Isofluran (3% (v/v), CP-Pharma) and sacrificed by cervical dislocation. The genotypes were confirmed with PCR using genomic DNA from tail biopsies with the following primers (forward: CTGCCAGGCATCTTTCTAGC; reverse: TTCTGCAGGTTGGAGGCAAC; neo reverse: CTGCTCTTTACTGAAGGCTC).

### Isolation and culture of adult neural stem cells from the murine subependymal zone

The following isolation protocol was adapted from a published procedure [8]. 9–11-week-old mice were sacrificed as described above. After decapitation, the brain was dissected and transferred into a 10 cm petri dish filled with ice-cold HBSS (#24020-091 Gibco, Darmstadt, DE) with 10 mM HEPES (#H0887-100 Sigma, Steinheim, DE), pH 7.4. The brain was split into its two hemispheres and cut coronally behind the optic chiasm with a surgical blade. Uncovering the ventricle from the caudal side using forceps, the lateral wall of the ventricle becomes visible. Ventricular wall tissue was dissected carefully and as thin a sheet as possible to avoid contamination with striatal tissue or myelin from the corpus callosum. The tissue was placed into a 15 ml tube containing 10 ml of fresh dissection medium (see above) until all brains were dissected. Afterwards, the medium was replaced by 5 ml of dissociation medium (HBSS with 15 mM HEPES and 0.54% (w/v) d-( +)-glucose (#D1349 AppliChem, Darmstadt, DE) supplemented with 6.7 mg trypsin (#T9201-100 Sigma) and 3.3 mg hyaluronidase (#H3884-50 Sigma) and incubated for 15 min at 37 °C. The tissue was gently triturated with a 5 ml pipette up to 10 times and then incubated for another 15 min at 37 °C. Tissue dissociation was stopped by adding the same volume of EBSS (#24010-043 Gibco) with 20 mM HEPES and 4% (w/v) BSA (#A9418 Sigma). The cell suspension was mixed gently before it was passed through a 70 µm cell strainer (#2350 Falcon). Cells were centrifuged at 200 g for 5 min. After aspirating the supernatant with a Pasteur pipette, cells were re-suspended in 10 ml of ice-cold HBSS with 0.9 M saccharose (#S/8600/60 Fisher Chemicals). Cells were centrifuged again for 15 min at 450 g. The resulting cell pellet was re-suspended in 2 ml ice-cold EBSS with 20 mM HEPES and 4% (w/v) BSA. 5 ml of the same solution were filled into a fresh 15 ml tube. The 2 ml cell suspension was gently applied to the top of the fresh solution. A final centrifugation step at 250 g for 9 min was carried out, followed by re-suspension of the pellet in culture medium containing DMEM/F12 (#11320-074 Gibco) with 1 × B27 (#17504-044 Gibco), 1 × penicillin/streptomycin (#P4333 Sigma) and 8 mM HEPES. The yield of one entire brain was seeded onto a poly-d-lysine (20 µg/ml, #P0899 Sigma) coated well of a 24-well culture plate. As optional treatment, 24-well culture plates were coated further with 10 µg/ml Laminin-1 (LN1) (#354259 Corning) or 25 µg/ml Tenascin-C (non-commercial, self-produced as described [38]). The cells were incubated at 37 °C and 5% (v/v) CO_2_.

### Time-lapse video microscopy

For the detailed investigation of adult neural stem cell proliferation behavior, the 24-well plate was placed into the closed system of an Axiovert 200 M equipped with an AxioCam HRm and AxioVision-4.8.1 software (all from Carl Zeiss, Oberkochen, DE). In addition, the two controlling elements ‘Tempcontrol 37-2 digital’ and ‘CTI-Controller 3700 digital’ (PeCon GmbH, Erbach, DE) were used for stable temperature and pH conditions, respectively. Conditions were adjusted 1 h before use. NSPCs were kept in culture medium (DMEM/F12 (#11320-074 Gibco) with 1 × B27 (#17504-044 Gibco), 1 × penicillin/streptomycin (#P4333 Sigma throughout the experiments. The absence of cytokines during the time-lapse studies was intended to uncover selective effects of the culture substrates and to focus on the intrinsic proliferation and differentiation behaviour of aNSPCs, as established in a previous study [8]. Over a period of 6 days, 8 visual fields per well were documented every 5 min with defined XYZ-coordinates controlled by a moving stage (Märzhäuser, Wetzlar, DE) to ensure a sharp focus over the whole period of time. Thereby, a stack from about 1680 single images can be combined to obtain a time-lapse video with detailed information about the cell behavior in vitro.

### Cell lineage tracking

To follow the lineage of a single mother cell, the obtained stack from time-lapse video microscopy was loaded into Fiji software. First, the visual field was scanned for proliferation events. When there was a proliferative cell, it was tracked from the beginning and each time point of cell division was noted with respect to the direct precursor. With this information, a lineage tree could be drawn that gave precise evidence for the cell cycle length of each generation. Each bifurcation in this tree means a divisional event and the length of the vertical lines corresponds to the duration of the observed cell cycle. Thus, each mother cell establishes its own individual lineage tree. Every dividing cell from five biologically independent experiments was included into the analysis (*N* = 5; wild type *n* = 497 and knockout *n* = 350).

### Migration analysis

Another read-out of the time-lapse videos focused on the migratory behavior of the generated neuroblasts. In most cases, neuroblasts arose from the final division of a mother cell in this culture system, which could be identified by a bipolar morphology and the gain of migratory properties. With Fiji software, cell movement could be evaluated using the ‘Manual Tracking’ Plugin. Parameters like distance (in arbitrary units, AU) and migration time (in min) were directly analyzed, while the speed parameter was calculated as distance per time (in AU/min).

### Neurosphere assay

To calculate the number of stem cells within the wild type and knockout SVZ, tissue was dissected and digested as described above and then seeded into T25 flasks with a density of 750–1000 cells/ml in DMEM/F12 medium supplemented with B27, penicillin/streptomycin, 8 mM HEPES and epidermal growth factor (EGF, 20 ng/ml, #100-15 PeproTech, Hamburg, DE). Without a coated surface, the stem cells grow in suspension and build neurospheres upon proliferation. After cells were incubated at 37 °C and 5% (v/v) CO2 and allowed to grow for 7 days, they were fixed by the addition of 1% (w/v) PFA into the culture medium. The total number of spheres per flask was counted under an inverted cell culture microscope with the help of a grid. As the cells were seeded in moderate density, one sphere was expected to originate from one stem cell and should not result from fusion events. To prevent cell clustering, any undue agitation of the cultivation medium was carefully avoided. Thereby, this method was used as an in vitro approach to determine stem cell numbers in both genotypes.

### mRNA expression analysis via RT-PCR

To isolate mRNA from the tissue, three pieces of lateral ventricular wall tissue were pooled for one sample and covered with 250 µl lysis buffer supplemented with 2.5 µl β-mercaptoethanol from the GenEluteTM Mammalian Total RNA Miniprep Kit according to manufacturer’s instructions (#RTN350 Sigma). An on-column DNAse digestion step was inserted (#DNASE70 Sigma). After elution with 30 µl H2O, RNA concentrations were measured with a photometer (Hellma Analytics). Purified mRNA was used for cDNA synthesis. If possible, 0.5 µg RNA was taken for one reaction. When concentrations were minute, the whole yield of mRNA was used into the synthesis reaction. Manufacturer’s instructions for the First Strand cDNA Synthesis Kit were followed (#K1612 Thermo Scientific). The RT-PCR method was used to analyze changes in gene expression levels between the wild type and Tnc knockout mice. The regulation was estimated in a semi-quantitative manner by setting amplicon levels in relation to a housekeeping gene that does not underlie regulation in the corresponding tissue. ß-actin was generally used as reference gene. For detailed information concerning the used primers please, see the supplementary material section (supplemental table S3).

### Protein expression analysis via western blot

For protein analysis, tissue was lysed with 100 µl of protein lysis buffer (50 mM Tris/HCl pH 7.4, 150 mM NaCl, 5 mM EDTA, 5 mM EGTA, 1% (v/v) Triton-X 100, 0.1% (v/v) Na-deoxycholate, 0.1% (v/v) SDS, 1 mM orthovanadate, 40 mM sodium fluoride) supplemented with 10 µl/ml PMSF (#195,381 MP Biomedicals) and 10 µl/ml Aprotinin (#10 981 532 001 Roche) as protease inhibitors. After mechanical trituration of the tissue, samples were incubated for 15 min on ice followed by centrifugation at 16,000 rpm for 15 min at 4 °C. The supernatant was transferred to a new tube and concentrations were determined with PierceTM BCA Protein Assay Kit (#23225 Thermo Scientific). A protein amount of 10 µg was loaded onto a 10% (v/v) polyacrylamide gel. Gels ran with a current of 15 mA for 1 h. Proteins were transferred to a PVDF membrane by applying a current of 75 mA for 1 h. Membranes were blocked with 5% (w/v) milk powder (Heirler, Radolfzell, DE) in TBS buffer (10 mM Tris/HCl pH 7.4 with 150 mM NaCl) for 2 h, before primary antibodies were diluted in blocking buffer and incubated overnight at 4 °C. After three cycles of washing with TBST (TBS with 0.05% Tween-20 (VWR Chemicals, Darmstadt, DE)), membranes were covered with secondary antibodies diluted in blocking buffer for 1 h and rotated on an orbital shaker. Before the blot was developed, membranes were washed again for three times with TBST and once with TBS to remove Tween residues. Afterwards, both solutions of the Clarity Western ECL Substrate (#1705061 BioRad) were mixed in equal parts and applied onto the membrane to incubate for 5 min. The solution was drained from the membrane to document chemiluminescent signals using the MicroChemi (DNR Bio-Imaging systems).

### Differentiation assay and immunocytochemistry

To reveal differences in the cell fate of adult neural stem cells driven by the different extracellular matrix components which they were exposed to, cells were incubated for 7 days at 37 °C and 5% (v/v) CO_2_. Within this time period, the progeny generated by stem cells in the culture was able to differentiate to neuroblasts (Dcx+), astrocytes (GFAP+) or oligodendrocytes (O4+). To stain the culture after 7 div, cells were fixed with 4% (w/v) PFA for 10 min. After three washing steps with PBS for 5 min, cells were incubated with the primary antibodies at 4 °C over night (mouse IgM anti-O4 (RRID: AB_94872; 1:30), rabbit anti-GFAP (RRID: AB_477010; 1:300) and mouse anti-bIII-tubulin (RRID: AB_477590; 1:300)) and 0.5% (v/v) Triton X-100 (#A4975 AppliChem). After three washing steps with PBS for 15 min each, secondary antibodies were diluted in the same buffer as primary antibodies and incubated for 1 h at room temperature (goat anti-mouse IgM AF488 (RRID: AB_2338849), goat anti-mouse IgG Cy3 (RRID: AB_2338686) and goat anti-rabbit IgG Cy5 (RRID: AB_2338013), all 1:300 from Dianova; supplemental table S4). Coverslips were placed in a drop of ImmuMount on an object slide with the cells facing downwards. Stainings were documented at an Axiophot (Zeiss).

### Tissue fixation and preparation of cryosections

For immunohistochemical stainings, the brain was taken out of the skull and directly stored in 4% (w/v) PFA at 4 °C for 48 h. This fixation was followed by a sucrose gradient: brains stayed at 10% (w/v) sucrose for 6 h, 20% (w/v) sucrose for 24 h and 30% (w/v) sucrose for another 24 h, before they were embedded in tissue freezing medium (Leica Microsystems, Wetzlar, DE) and placed onto dry ice to ensure fast freezing of the tissue. Cryosections were cut at the Leica Cryostat CM3050S with 14 µm thickness.

### Immunohistochemistry

Sections were thawed and allowed to dry. The tissue was encircled with a hydrophobic barrier marker (ROTI ®Liquid Barrier marker, Carl Roth, Karlsruhe, DE). Sections were rehydrated in PBS with 1.7% (w/v) NaCl (Fisher Chemical) for 1 h. Afterwards, sections were covered for 2 h with blocking buffer containing 10% (v/v) normal goat serum (Dianova), 1% (w/v) BSA (Sigma) and 0.1% (v/v) triton X-100 (AppliChem) in PBS. Primary antibodies were diluted in blocking buffer and incubated overnight at 4 °C. After three washing steps with PBS for 15 min each, secondary antibodies were diluted in blocking buffer and applied to the sections for 2 h at room temperature. Another three cycles of washing with PBS were conducted before the sections were covered with ImmuMount (Thermo Scientific) and a 24 × 50 mm coverslip. Stainings were documented at the AxioZoom V16 (Carl Zeiss).

### Statistical analysis

Results in bar charts are depicted as mean ± s.e.m., unless otherwise specified. Results that are presented as Box–Whisker-Plots show the median as line, the upper and lower quartile as box and the 5–95% percentile as whiskers. The statistical test and the number of performed experiments are indicated in the figure legends, where biological replicates are referred to as “*N*” and technical replicates are referred to as “*n*”. The Kolmogorov–Smirnov test was used to verify Gaussian distribution. Statistical tests were done with the GraphPad Prism 7 software (Graphpad Software, San Diego, CA). Significances were graphically illustrated by * for *p* < 0.05, ** for *p* < 0.01 and *** for *p* < 0.001.

## Results

### Expression pattern of Tnc in the adult murine brain

**Fig. 1:**
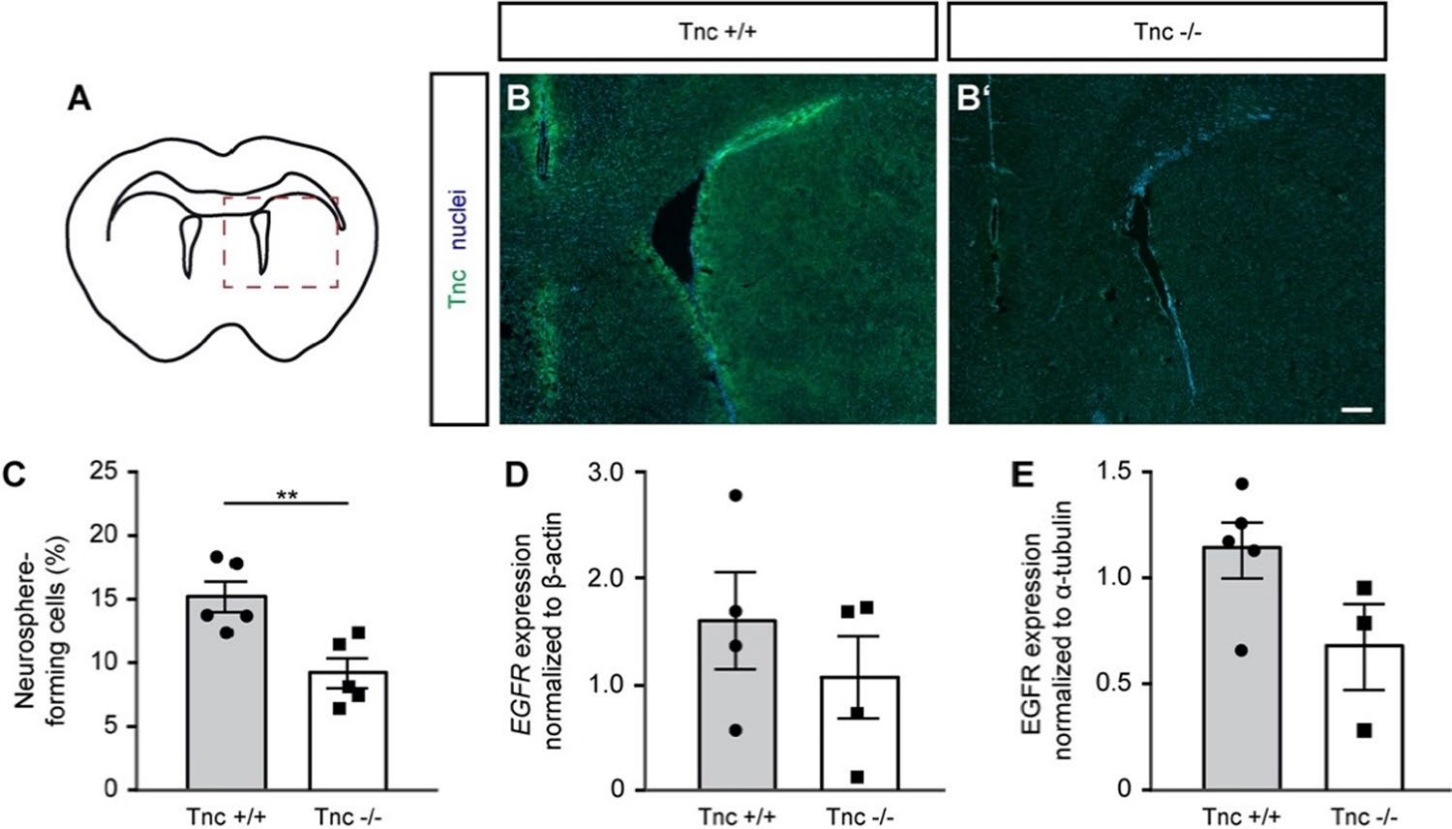
Adult neural stem cells isolated from Tnc-deficient mice exhibit a reduced neurosphere-formation capacity in vitro. The scheme depicted in **a** shows the contour of an adult brain in frontal sections with the two triangularly formed lateral ventricles and the curved structure of the corpus callosum above them. The red dotted line marks the detail that is illustrated in the immunohistochemical staining in **b**, **b′**. In the control (**b**), the distribution of the Tnc glycoprotein at the age of P70 can be detected around the lateral ventricle as well as around the third ventricle by polyclonal anti-Tnc antibodies. **b′** shows the same staining in a Tnc-deficient animal. There is no fluorescent signal detectable, which supports the specificity of the antibody and confirms the efficient knockout. Scale bar: 200 µm. **c** Adult neural stem/progenitor cells isolated from 10-weeks old mice were cultivated in presence of 20 ng/ml EGF. The percentage of plated cells generating neurospheres after 7 days in vitro is significantly reduced in Tnc-knockout mice (*N* = 5). An unpaired two-tailed Student’s *t* test was assessed for the statistics (**p < 0.01). (D) RT-PCR analysis of isolated SEZ tissue showed a reduced expression of the required EGF receptor on mRNA level in vivo (*N* = 4). **e** The PCR result is supported by Western blot analysis of the EGF receptor, which verified a trend towards reduced expression also on the protein level (wild type *N* = 5, Tnc knockout *N* = 3). Data are presented as mean ± s.e.m. For **d**–**e**, the Mann–Whitney *U* test was used for statistical analysis

In the course of murine development, Tnc expression reaches a maximum right before birth to be strongly reduced afterwards [21]. Nevertheless, an immunohistochemical staining of frontal sections from wild-type mice at the age of 10 weeks (Fig. 1a) with polyclonal anti-Tnc revealed a restricted but distinct expression of Tnc around the lateral and the third ventricle (Fig. 1b). The specificity of the antibody was confirmed by controls with Tnc-deficient brain sections where staining was completely absent (Fig. 1b′). The close association of the secreted Tnc protein with stem cell niches in embryos that is maintained in the adult stage led to the assumption that this ECM component provides a beneficial environment for stem cell maintenance [28, 39].

### Tnc-deficient aNSPCs display a reduced response to EGF in vitro

To probe the asserted functional role, a classical experimental approach was used to determine differences in aNSPC numbers between wild type and Tnc-deficient mice in vitro. The neurosphere assay was performed with single cells isolated from the lateral ventricular wall that were cultivated in suspension cultures in presence of the cytokine epidermal growth factor (EGF). Expanding neurospheres did not form in the absence of the cytokine. After 7 days in vitro, the number of generated neurospheres was evaluated relatively to the sum of plated cells. Cells were seeded in a moderate density (700–1000 cells per ml) to ensure that each neurosphere results from the progeny of a single stem/progenitor cell (Supplemental Fig. S1). Tnc-deficient cells produced significantly less neurospheres after 7 days than wild-type cells (15.35% ± 1.2% neurosphere-forming cells in wild-type cultures compared to 9.32% ± 1.2% in knockout cultures, *N* = 5, *p* = 0.007 in unpaired Students *t* test, Fig. 1c).

The reduced capacity to form neurospheres could result from changes of EGF receptor (EGFR) expression. Therefore, the EGFR level in SEZ tissue in vivo was analyzed using PCR and Western blot. The expression of EGFR on mRNA level normalized to β-actin showed a decrease in Tnc-deficient animals (1.06 ± 0.39 compared to 1.60 ± 0.46 in wild types, mean ± s.e.m., *N *= 4, Fig. 1d). The protein expression paralleled this result, as Tnc-deficient tissue seemingly displayed reduced EGFR levels compared to the control (0.67 ± 0.20 in knockout SEZ (*N* = 3) compared to 1.13 ± 0.13 in wild-type SEZ (*N* = 5); however, not significant, *p* = 0.14 in Mann–Whitney *U* test, Fig. 1e). In conclusion, the aNSPC population of the SEZ of Tnc-knockout mice exhibited a tampered proliferative capacity in response to EGF. This deficit has not been observed in an earlier study of the adult *Tnc*^*−/−*^ aNSPCs, presumably because the cells had been cultivated in a medium containing both 20 µg/ml EGF and FGF2 [40]. FGF2 by itself is known to promote proliferation of NSPCs and the expression of the EGFR, effects that apparently overrode the delayed responsivness to EGF observed here [41]. In our view, the reduced responsiveness to EGF accounts for the overall reduced number of neurospheres, although Tnc-deficient cells revealed a shorter cell cycle (see below). The role of the EGFR in this context remains to be established.

### Cell lineage tracking of adult NSPCs by time-lapse video microscopy permits measurement of cell cycle length

**Fig. 2:**
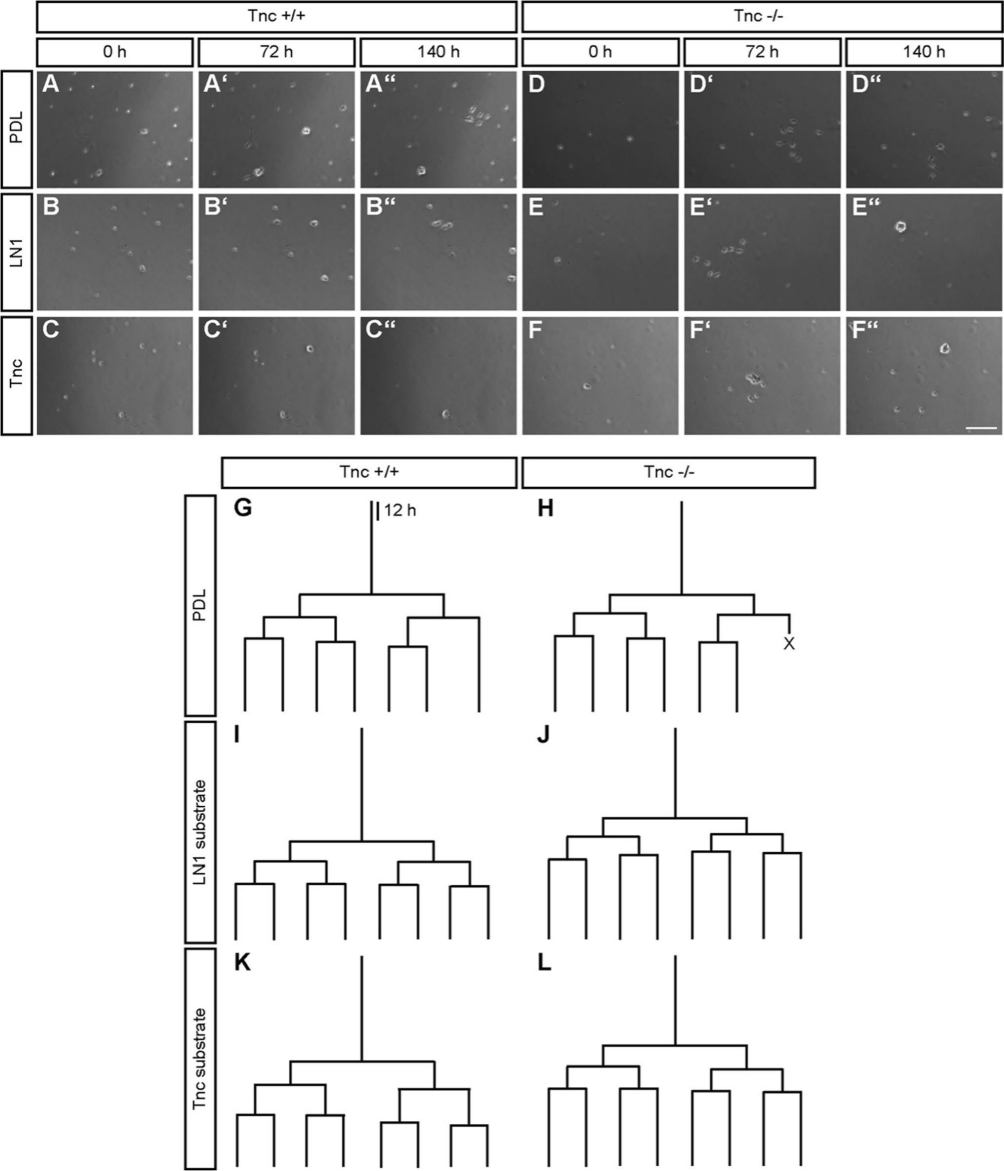
Phase contrast images and exemplary lineage trees of wild type and Tnc-deficient SEZ cells on three experimentally tested substrates using time-lapse video microscopy over 6 div. For each condition, the time series depicts the identical field of view. **a**–**c** Phase contrast images of wild-type cells in all three conditions. The cells generally showed good adherence and survival rates. Proliferation events were visible after 72 h, when first cell assemblies could be observed. After 140 h, numerous cells appeared branched. Cultivation on Tnc coated PDL also led to cell proliferation. However, increased cell loss and the tendency to cell aggregation with diminished adhesion to the culture plate were observed in this condition. **d**–**f** Tnc-deficient cells also showed satisfying adhesion in the control condition and fast proliferation within 72 h. In the next three days, cells on LN1 and Tnc started to cluster, which reduced the adhesion to the culture plate (**eʺ** and **fʺ**). Scale bar: 150 µm. **g**–**l** Exemplary pedigrees of individual mother cells in each condition monitored by lineage tracking are shown. Each lineage tree traces the proliferation of an initial mother cell to map the whole progeny that was generated within 140 h of time-lapse video microscopy. Every branching point marks a cell division event and the length of the branches is proportional to the corresponding cell cycle duration. An ‘X’ marks the death of a daughter cell

A detailed evaluation of cell cycle duration in wild type and Tnc-deficient aNSPCs was conducted with the help of video time-lapse imaging over a period of 6 div. The single cells were plated onto PDL coated control dishes or to substrates additionally replenished with laminin-1 (LN1) or tenascin-C (Tnc). The subependymal cells from one embryonic brain were plated per singular Costar well and cytokines were omitted to focus on the intrinsic proliferation and differentiation properties of NSPCs. Under these conditions, aNSPCs and their progeny can be observed in culture for up to 6 days [8]. On average, 102 ± 14 mitotic cells were detected per video in wild type compared to 82 ± 12 mitotic cells per knockout culture. Each aNSPC mother cell constructed its own individual pedigree depending on the number of divisions and the length of the cell cycle (exemplary lineage trees for each condition are mapped in Fig. 2g–l). A striking attribute of lineage trees generated by aNSPCs was their synchronized sequence (Supplemental movies 1–4). Daughter cells mostly divided again after comparable cycle lengths. Note that the mapped lineage trees all showed three rounds of division. This happened in 10% of the mother cells (data not shown), but served better for illustration of the proliferation dynamics. Most of the generated trees were slenderer and displayed fewer branching points. Individual pedigrees differed with regard to the number of divisions and the occurrence of asymmetric versus symmetric division modes. In about 50% of the cases we recorded one, in 33% two and in 10–15% three ore more division rounds during the observation period (compare also supplemental Fig. S2). The mapping provided a good overview of the sequential divisions of a single mother cell, but it did not yield sufficient information that permitted precise quantification and statistical evaluation. For this reason, the complete data set relating to cell cycle lengths was assembled in Box–Whisker-plots.

### Tracking of adult NSPCs by time-lapse video microscopy reveals differences between Tnc and LN1 substrates

**Fig. 3:**
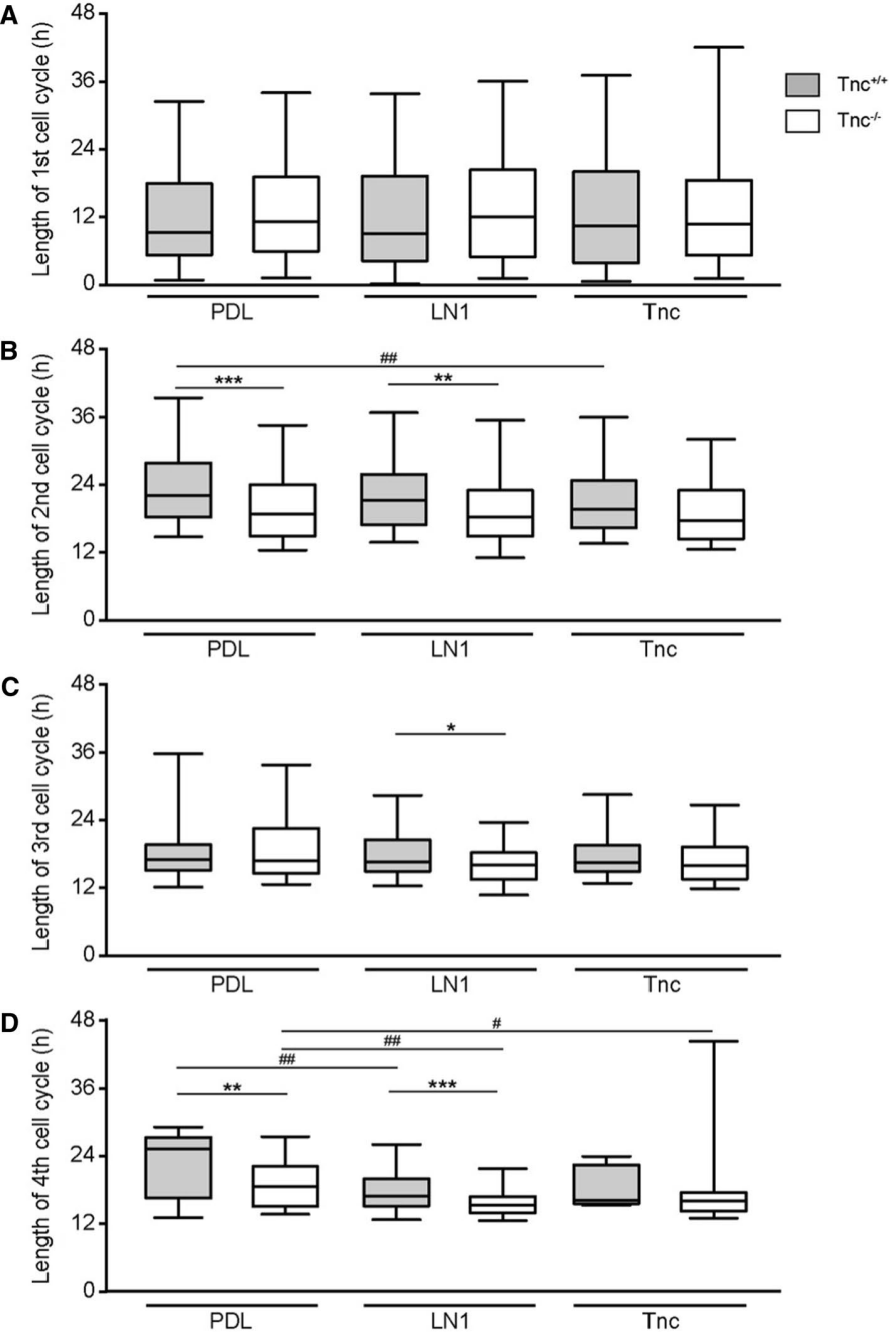
Cell cycle length of proliferative populations is reduced in Tnc-deficient SEZ cultures. **a** Cell cycle length of the 1st generation in vitro. Please note that in this special case only the endpoint could be determined, resulting in a broader scattering of the data points. **b** Graphs display the results for the 2nd generation in vitro. The cell cycle of wild-type aNSPCs was significantly accelerated on Tnc coated culture dishes. Tnc-deficient cells divided faster on the PDL control and on LN1, but not on Tnc substrate. **c** Illustrates results for the 3rd generation in vitro where cell cycle lengths were reduced in Tnc-deficient cells on the LN1 substrate compared to the wild type. **d** Represents the results of the 4th generation in vitro. Wildtype cells displayed a shorter cell cycle on LN1, whereas knockout cells divided faster on both LN1 and Tnc. A significantly accelerated cell cycle was also observed in Tnc-knockout cells in comparison to their wild type equivalent in the control condition and on the LN1 substrate. Five biologically independent experiments were performed (*N* = 5). Every observed cell division in each video was included in the analysis (*n* = 7–185). More detailed information concerning the data set is available in supplemental table 2. Data are presented as box-whisker-plots with 5–95% percentiles. The Kruskal–Wallis test with Dunn’s multiple comparison post-test was used for statistical analysis of differences between the substrates (*p < 0.05, **p < 0.01). The Mann–Whitney *U* tests were performed for statistical analysis between the genotypes (*p < 0.05, **p < 0.01, ***p < 0.001)

In a first approach, the total proliferative cell population was included in the analysis. At this initial stage of our study, no obvious subpopulations were distinguished. Individual dividing cells were chosen and monitored over a period of up to 6 days. Previous studies using time-lapse video microscopy tracking already revealed significant differences in the cell cycle lengths of subsequent generations of embryonic spinal cord NSPCs [8, 29]. Therefore, each generation was considered separately. The first generation represented a peculiar condition due to the fact that the beginning of the cycle could not be determined with precision as it laid outside of the observed time frame. This accounted for a broader distribution of the data points and no significant differences were hence observed for the first generation of wild type aNSPCs between PDL (median of 9.7 h, *n* = 171, *N* = 5), LN1 (9.5 h, *n* = 185, *N* = 5) and Tnc (10.8 h, *n* = 141, *N* = 5; Fig. 3a) conditions. Knockout cells showed medians of 11.3 h, 12.2 h and 10.9 h on PDL (*n* = 150, *N* = 5), LN1 (*n* = 107, *N* = 5) and Tnc (*n* = 93, *N* = 5, Fig. 3a), respectively.

In the second generation, a significant decrease of the cycle length from 21.8 h (*n* = 157, *N* = 5) in the control to 19.3 h on Tnc (*n* = 145, *N* = 5, *p* = 0.002, Fig. 3b) was found in wild-type cells. Cell cycle length on the LN1 coating was unaltered, with 21.0 h (*n* = 176, *N* = 5). No changes were detected between the three conditions in the second generation of knockout aNSPCs (PDL: 18.7 h, *n* = 117, *N* = 5; LN1: 18.1 h, *n* = 106, *N* = 5; Tnc: 17.6 h, *n* = 103, *N* = 5; Fig. 3B). An analogous outcome occurred in the third generation of wild-type aNSPCs (PDL: 17.2 h, *n* = 81, *N* = 5; LN1: 16.8 h, n = 125, *N* = 5; Tnc: 16.6 h, *n* = 67, *N* = 5) and Tnc-knockout cells (PDL: 17.0 h, *n* = 106, *N* = 5; LN1: 16.3 h, *n* = 79, *N* = 5; Tnc: 16.1 h, *n* = 96, *N* = 5; Fig. 3C). The fourth tracked generation, however, revealed a significantly shorter cell cycle in wild-type aNSPCs on the LN1 substrate (17.0 h, *n* = 57, *N* = 5; Fig. 3d) compared to the control (25.3 h, *n* = 21, *N* = 5, *p* = 0.004). The median cell cycle on the Tnc substrate was similarly reduced to 16.3 h, but this result did not achieve statistical significance due to a small sample size (*n* = 7, *N* = 5). The accelerated division cycle consequent to a reduced cycle length in the presence of ECM constituents also emerged in knockout aNSPCs, where it proved significant for both substrate conditions. On LN1 aNSPCs displayed a median of 15.0 h (*n* = 29, *N* = 5, *p* = 0.005, Fig. 3d) and on Tnc a median of 15.8 h (*n* = 52, *N* = 5, *p* = 0.040), compared to 18.3 h in the PDL control (*n* = 54, *N* = 5). In conclusion, the exposure to ECM substrates like LN1 or Tnc caused an acceleration of the cell cycle of aNSPC in vitro (Supplemental table 1).

### Cell lineage tracking of adult NSPCs by time-lapse video microscopy reveals faster cell cycling in Tnc-knockout cultures

So far, the study was focused on the effect of supplemented ECM-glycoproteins presented as substrates in culture. It is also of interest to consider the behavior of wild type and knockout aNSPCs under identical culture conditions to evaluate the relative contribution of Tnc genes. Because of the impossibility to fix the time point of the first division mentioned previously, no significant changes could be traced in the first generation (Fig. 3a). In the second generation, Tnc-deficient aNSPCs cycled significantly faster on PDL (18.7 h, *n* = 117, *N* = 5, Fig. 3b) compared to wild-type aNSPCs (21.8 h, *n* = 157, *N* = 5, *p* < 0.001). An analogous phenomenon was also seen on LN1 (wild type: 18.1 h, *n* = 106, *N* = 5; knockout: 21.0 h, *n* = 176, *N* = 5, p = 0.003). The result on the Tnc substrate was not significant in the second generation (wild type: 19.3 h, *n* = 145, *N* = 5; knockout: 17.6 h, *n* = 103, *N* = 5, p = 0.11; Fig. 3b). In the third generation, cell cycle lengths did not show significant alterations on PDL (wild type: 17.2 h, *n* = 81; *N* = 5; knockout: 17.0 h, *n* = 106, *N* = 5) and Tnc (wild type: 16.6 h, *n* = 67; *N* = 5; knockout: 16.1 h, *n* = 96; *N* = 5, p = 0.15; Fig. 3c). On LN1, the decrease in cell cycle length of Tnc-knockout cells was significant (16.3 h, *n* = 79; *N* = 5; Fig. 3c) compared to the wild type (16.8 h, *n* = 125, *N* = 5, *p* = 0.041). The situation in the fourth generation was comparable in that Tnc-deficient aNSPCs (18.3 h, *n* = 54, *N* = 5) divided faster than the wild type on PDL (25.3 h, *n* = 21, *N* = 5, *p* = 0.007; Fig. 3d). The outcome was comparable on the LN1 substrate (knockout: 15.0 h, *n* = 29, *N* = 5; wild type: 17.0 h, *n* = 57, *N* = 5, *p* < 0.001; Fig. 3d). On the Tnc coating, however, the difference was not significant (knockout: 15.8 h, *n* = 52, *N* = 5; wild type: 16.3 h, *n* = 7, *N* = 5, *p* = 0.12; supplemental table S1). Taken together, both the addition of Tnc as culture substrate and, alternatively, its depletion by genetic recombination in the knockout resulted in alterations of the cell cycle length in aNSPCs. Based on these results we proposed the hypothesis that the Tnc concentration is subtly balanced in the niche. Consequently, its dysregulation unsettled the proliferation behavior of aNSPCs in vitro. On the other hand, the responsivness of Tnc-deficient aNSPCs to growth factors appeared reduced, which may have counterbalanced the shortened cell cycle and resulted in a net reduction of neurospheres (Fig. 1).

#### Identification of a subpopulation of slowly dividing aNSPCs using time-lapse video microscopy

**Fig. 4:**
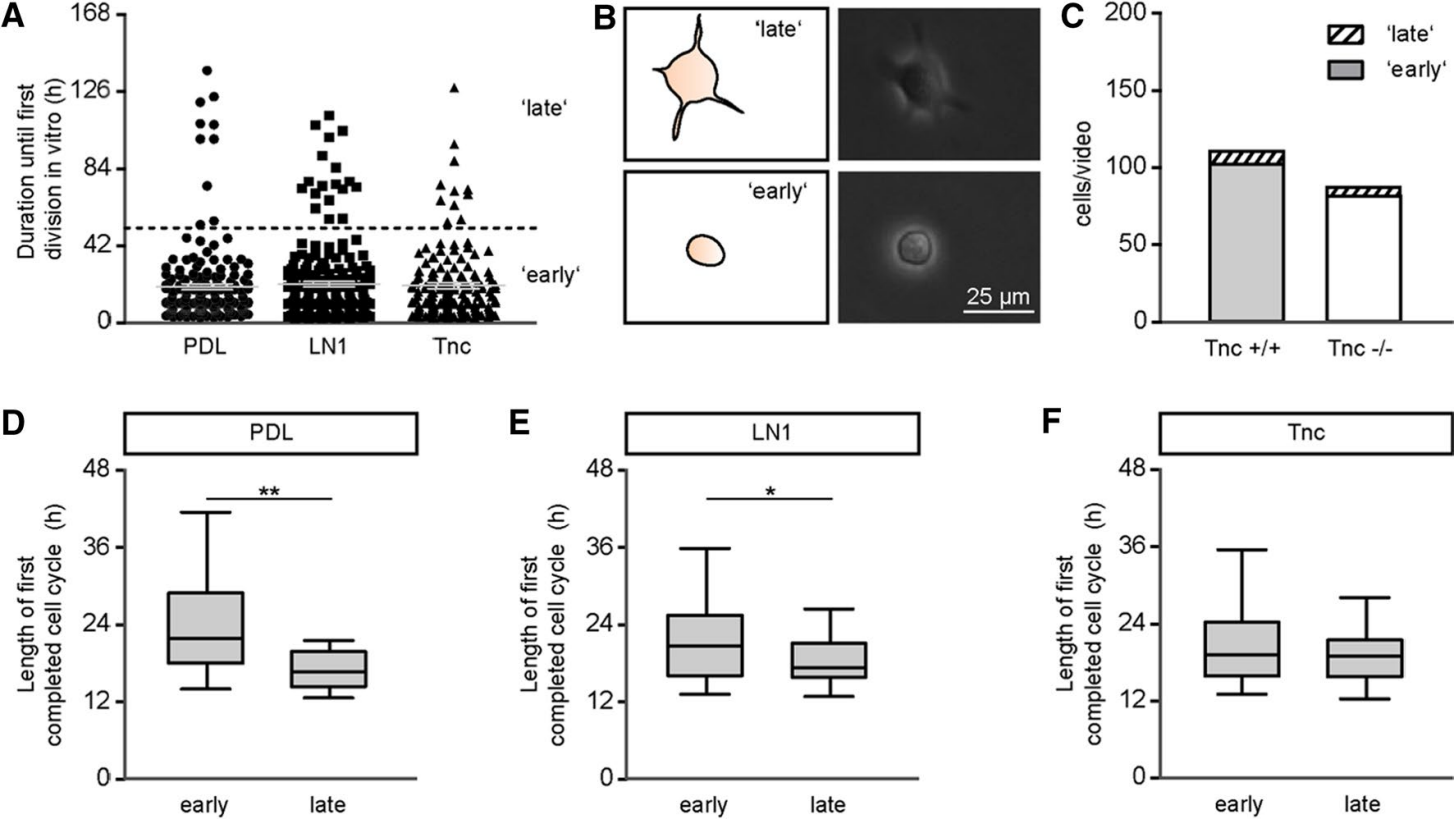
Late-dividing stem cells are a veritable subpopulation in primary SEZ cultures. **a** The graph presents the duration until the first division in vitro for every mother cell of the wild type dataset, separately plotted for the three substrate conditions PDL (*n* = 171, *N* = 5), LN1 (*n* = 185, *N* = 5) and Tnc (*n* = 141, *N* = 5). Gray lines represent mean ± s.e.m. The scatter of data points revealed a gap around 50 h of documentation. This boundary was used to define a threshold (black dotted line) that separates a subset of 10% of the dividing cells from the majority located underneath the threshold. **b** A morphological examination of both populations presented as scheme (left) and in phase contrast images (right) indicates that the ‘late’-labelled aNSPC_late_ subpopulation possesses larger cell bodies with outgrowing branches (top) in comparison to the ‘early’-labelled mitotic aNSPC_early_ cells with a phase-bright round cell body (bottom). Scale bar: 25 µm. **c** Bar chart illustrating the diverging fractions of early- and late-dividing cells in wild type and Tnc-deficient cultures. Independently of the genotype, late-dividing aNSPCs_late_ represented a minority of less than 10% of the dividing cells in the SEZ cultures. **d**–**f** Comparing the length of the first completed cell cycle between early- and late-dividing wild type cells indicated a significant decrease in the cell cycle of late-dividing cells on PDL (**d**) and LN1 (**e**), but not on Tnc (**f**). The Mann–Whitney *U* test was used for statistical analysis (*p < 0.05, **p < 0.01)

When the proliferation activity of aNSPCs was examined in more detail, a subset of proliferating cells appeared to engage remarkably late into cell division during the observation period of 6 days. This subset remained notably inert, while the majority of proliferating cells performed two rounds of mitosis. This resulted in a remarkable extension of the temporal delay until the first round of mitosis that was clearly visible when the temporal sequences until the first division were plotted for the individual cells monitored in the cell tracking experiment (Fig. 4a). Whereas the majority of proliferating cells started their first division within the first 24 h of tracking, a minority stalled for up to 48 h. In order to distinguish this subpopulation, a threshold was set at 50 h, following the assumption that a subclass of slowly dividing aNSPCs would cluster above this hypothetical barrier. This procedure was supported by the observation that early dividing aNSPCs exhibited a different morphology compared to late dividing ones (Fig. 4b). The rapidly dividing cells were characterized by a rounded small cell body, while the late dividers presented several elongated processes emanating from a cell body that was nearly twice as large (top). An exemplary scheme of both cell morphologies was added to the phase contrast images. The durations of the first completed cell cycle were compared for the rapidly dividing as compared to the late-dividing population. On PDL, the groups differed significantly as early dividing cells needed 21.8 h (median, *n* = 157, *N* = 5; Fig. 4d) while the late subtype displayed a faster division (16.8 h, *n* = 10, *N* = 5, *p* = 0.009). On LN1, the difference was comparable (early dividing: 21.0 h, *n* = 176, *N* = 5; late dividing: 17.5 h, *n* = 24, *N* = 5, *p* = 0.03; Fig. 4e). In this context culturing on Tnc substrates proved an exception, because both early and late dividing cells displayed comparable cycling times (early dividing: 19.3 h, *n* = 145, *N* = 5; late dividing: 19.2 h, *n* = 27, *N* = 5; Fig. 4f; supplemental table S2). The proportion of early to late dividing cells was 102.2 versus 8.2 cells per video (mean, *N* = 5) for wild type cultures and 81.6 versus 5.8 cells per video for knockout cultures (Fig. 4c). We hypothesize that the late dividing aNSPCs correspond to the slow-dividing astro/radial glia that has been described previously [9]. This cell type is placed at the top of a lineage tree and generates complex pedigrees. Most of these trees are characterized by the occurrence of asymmetric cell divisions and give rise to neuronal as well as astroglial progeny. In this sense they have been perceived as genuine aNSPCs, different from already fate-restricted progenitors [8].

The subpopulation will heretofore be referred to as aNSPCs_late_. In view of the small sample of aNSPC_late_ cells, we could not systematically test their lineage trees and differentiation behaviour. This subpopulation was included in the initial studies of cell proliferation (Fig. 3), but as it represents at most 10% of the collective under study, we do not think that it substantially skewed our results regarding cell-cycle length.

#### Examination of cell cycle lengths from the late-dividing subpopulation of aNSPCs

**Fig. 5:**
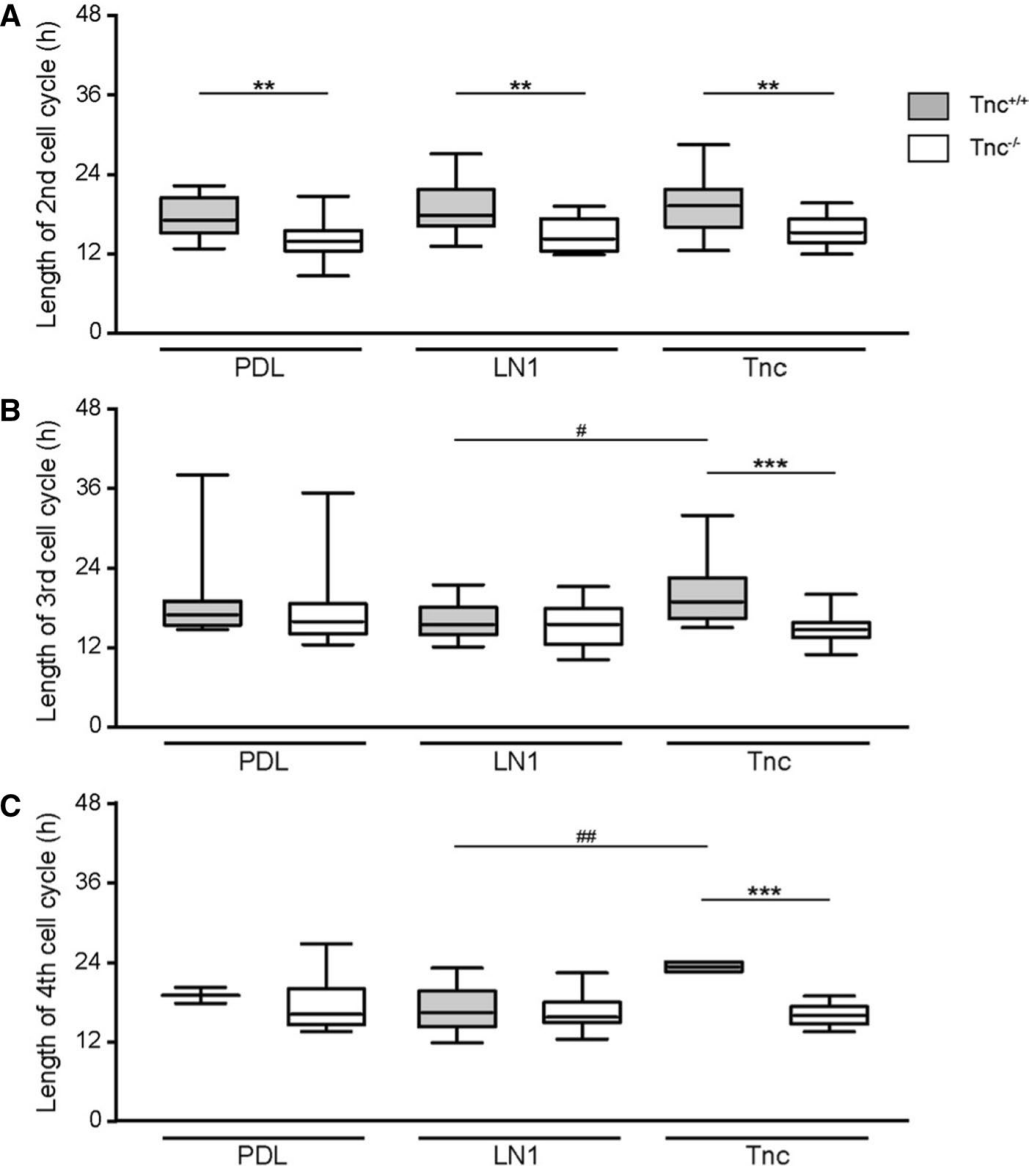
Cell cycle lengths of the wild type and Tnc-knockout subset of slow-dividing aNSPC_late_. **a**–**c** Cell cycle lengths of late-dividing wild type (gray) and knockout aNSPC_late_ (white) are presented in box-whisker-plots with 5–95% percentiles from the 2nd (**a**), 3rd (**b**) and 4th (**c**) generation (*N* = 5, *n* = 2–37). **a** In the 2nd generation, the cell cycle length was reduced in Tnc-deficient cells in the control, on LN1 and on Tnc substrates compared to their wild type counterpart. **b** Exposure to Tnc as substrate led to a significantly elongated cell cycle in the 3rd generation of wild type cells compared to the LN1 substrate. **c** This effect was maintained up to the 4th generation, which underscored the opposite effect of the Tnc substrate on the slow-dividing subset of aNSPC_late_ in comparison to the majority of the proliferative population. In Tnc-deficient aNSPC_late_, we could not detect differences between the different substrate conditions in any generation. Extensive information concerning the number of analyzed cells is presented in supplementary table S3. Statistical evaluation was conducted using the Kruskal–Wallis test with Dunn’s multiple comparison for comparing substrates (*p < 0.05, **p < 0.01). The Mann–Whitney *U* tests were performed to analyze differences between wild type and knockout data (**p < 0.01, ***p < 0.001)

The aNSPCs_late_ subpopulation defined by a prolonged inactive phase during the first 50 h of cell tracking was analyzed separately in more detail with regard to the behavior on different substrates (Fig. 5). To this end, the median values of time sequences of division in the different generations monitored were compared. In the second generation only minor differences on LN1 and Tnc were visible for wild type (median on PDL: 16.8 h, *n* = 10, *N* = 5; LN1: 17.5 h, *n* = 24, *N* = 5; Tnc: 19.2 h, *n* = 27, *N* = 5; Fig. 5a) and *Tnc*^*−/−*^ aNSPCs_late_ cells (median on PDL: 13.9 h, *n* = 20, *N* = 5; LN1: 14.0 h, *n* = 14, *N* = 5; Tnc: 15.2 h, *n* = 16, *N* = 5; Fig. 5a). In the third generation, however, the cell cycle length of wild type aNSPCs_late_ was significantly extended on Tnc compared to LN1 (18.8 h, *n* = 24, *N* = 5; versus 16.5 h, *n* = 32, *N* = 5, respectively, *p* = 0.046; Fig. 5b), while a median of 17.2 h was noted on PDL (*n* = 9, *N* = 5). Interestingly, no apparent differences were measured for *Tnc*^*−/−*^ aNSPCs_late_ in the third generation (PDL: 15.9 h *n* = 37, *N* = 5; LN1: 15.3 h, *n* = 26, *N* = 5; Tnc: 14.7 h, *n* = 23, *N* = 5). In the fourth generation, the observed difference with wild type aNSPCs_late_ was reiterated in that the cycle length was stretched to 23.3 h (*n* = 4, *N* = 5) on Tnc compared to 16.3 h (*n* = 22, *N* = 5) on LN1 (*p* = 0.001, Fig. 5c). In obvious contrast, cell cycle lengths of *Tnc*^*−/−*^ aNSPCs_late_ remained largely unchanged in the fourth generation (PDL: 16.0 h, *n* = 36, *N* = 5; LN1: 15.7 h, *n* = 18, *N* = 5; Tnc: 16.0 h, *n* = 22, *N* = 5, Fig. 5c). It is of interest to point out that the overall aNSPC population responded with an acceleration of proliferation on Tnc, whereas solely the wild type aNSPC_late_ subclass displayed a contrary behavior, namely protraction of the cell cycle. This provides further support for the argument that these represent two biologically different populations.

Remarkably, the prolongation of the cell cycle on Tnc was not seen with knockout aNSPCs_late_ (Fig. 5b, c). Thus, the mutant aNSPC_late_ did not respond with an elongation of the cell cycle to Tnc, in this regard different from the wild type cells (Fig. 4f). This may indicate that the *Tnc*^*−/−*^ cells may have lost sensitivity to the ECM component. The asserted differences between wild type and *Tnc*^*−/−*^ aNSPCs_late_ were followed further by direct comparison of the respective cell cycle lengths under the different conditions tested. Thus, in the second generation the knockout cells manifested a significant decrease of the cell cycle lengths on all substrates (*p* < 0.01 for each condition; Fig. 5a). In the third generation, no statistically significant differences could be discerned on the substrates PDL and LN1. In contrast, on Tnc the stretched cell cycle in the wild type compared to *Tnc*^*−/−*^ aNSPCs_late_ was obvious (wild type: 18.8 h, *n* = 24, *N* = 5; knockout: 14.7 h, *n* = 23; *N* = 5, *p* < 0.001; Fig. 5b). In the fourth generation the situation was analogous to the third generation. In particular, the significantly reduced cell cycle length of Tnc-deficient cells on a Tnc substrate persisted into the fourth generation (Fig. 5c). Taken together, this analysis indicated that *Tnc*^*−/−*^ aNSPCs_late_ deriving from Tnc-deficient stem cell niches could not respond to distinct ECM signaling cues in the same manner as their wild-type counterparts.

#### Functional analysis of aNSPC progeny in wild type and Tnc-deficient cultures

**Fig. 6:**
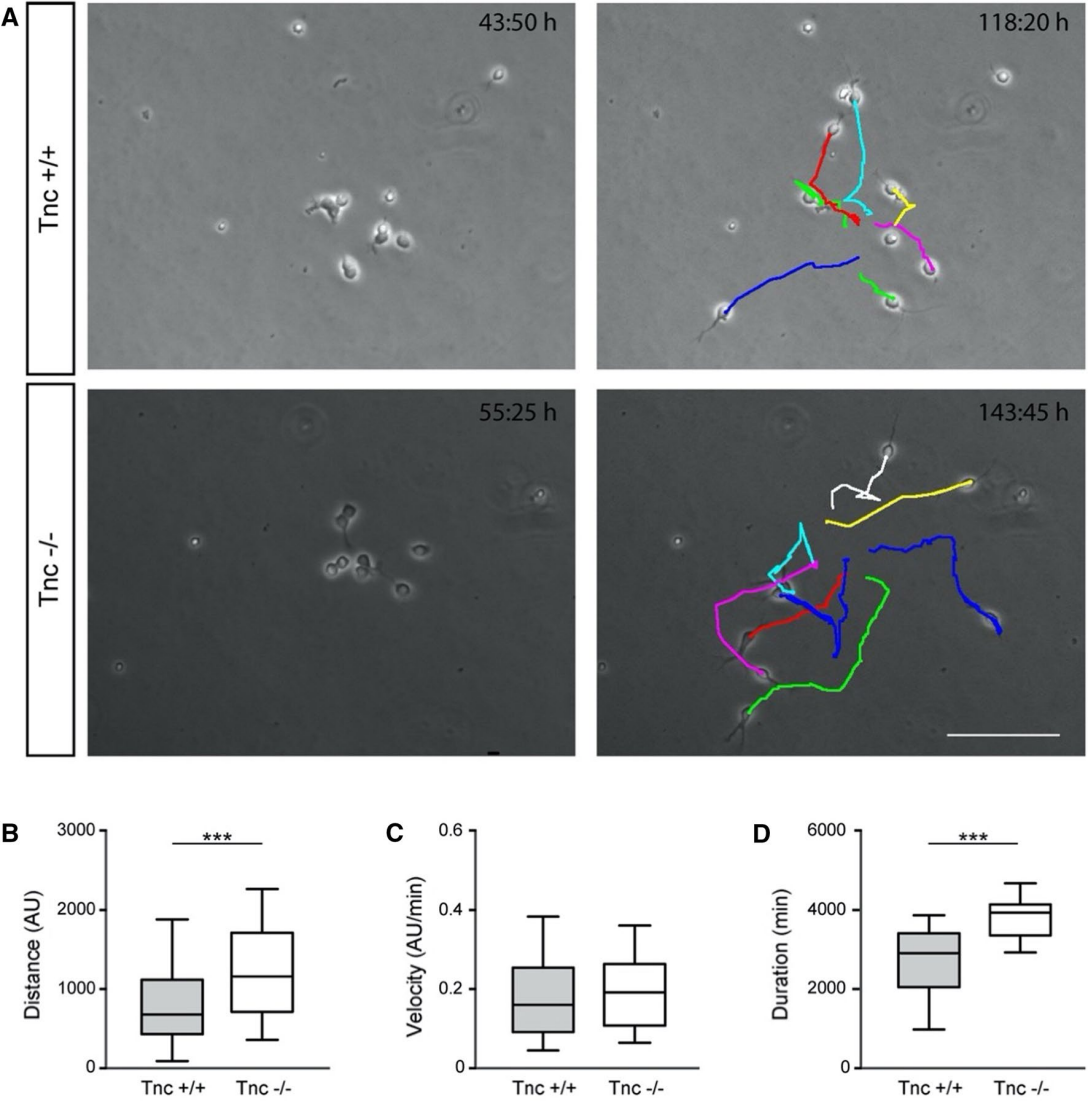
Neuroblast migration is increased in Tnc-deficient cultures. **a** Phase contrast images of wild type and Tnc-knockout aNSPCs on PDL substrate illustrate the generation of neuroblasts during time-lapse video microscopy (left) and the tracks of their migration (right), evaluated with the manual tracking plugin of FIJI software. Please note the individual time stamp within each micrograph. Scale bar: 100 µm. **b** Bar chart presenting the covered distance in arbitrary units (AU). Tnc-deficient cells cover a significantly longer distance compared to the control (*N* = 3, *n* = 32). **c** Analysis of the velocity in AU per minute reveal that the observed changes in distance are not caused by a higher velocity of Tnc-knockout cells, as the speed range is equal in both groups (*N* = 3, *n* = 32). **d** Evaluation of the temporal sequence (min) shows a significant increase of the duration of migratory phases for Tnc-deficient compared to wild type neuroblasts. Duration is measured from initiation of migration to the moment the neuroblast arrests and remains immobile (*N* = 3, *n* = 32). Data are presented as mean ± s.e.m. The unpaired two-tailed *t*-test was used for statistical analysis (***p < 0.001)

In the adult subependymal zone aNSPCs give rise to transient amplifying precursor cells and, eventually, to neuroblasts [17]. The latter migrate to the olfactory bulb where they differentiate to dopaminergic interneurons [42]. When the generation of neuroblasts was rated in our culture system, the incidence after 6 div ranged between 11.6 ± 7.0% (mean ± SD, *N* = 3) and 16.3 ± 3.7% (*N* = 3) in wild type and knockout cultures, respectively. The rather low frequency of neuroblasts may have resulted from the relatively short observation period of 6 days. The majority of stem cells thus may not have reached the end of their respective pedigrees. In order to obtain insight into the biological properties, the migratory activities of wild type and knockout neuroblasts were compared in the PDL control condition. They could be distinguished in our system because the more undifferentiated states (aNSPC or TAP) were static and did not display a bipolar morphology. Neuroblasts were tracked in phase contrast images on PDL from the initial to the last movement of the time-lapse video recordings (*n* = 34, *N* = 3; Fig. 6a). The time stamp indicates the temporal dimension as this factor is individual for the progeny of each lineage. The aNSPCs barely migrated as long as they were in a proliferative mode. The end point for movement analysis was defined as the moment when the neuroblast rested in a steady position and stopped migration. Wild-type cells covered a distance of 1522 ± 156 arbitrary units (AU, *n* = 34, *N* = 3), while Tnc-deficient neuroblasts achieved a path length of 2418 ± 204 AU (*n* = 32, mean ± s.e.m., *N* = 3, *p* < 0.001, Fig. 6b). In theory, longer distances could be caused by a higher velocity or by longer duration of the motile activity. The velocity, however, was not different between both genotypes (wild type: 0.45 ± 0.04 AU/min, *n* = 34, *N* = 3; Tnc-knockout: 0.47 ± 0.04 AU/min, *n* = 32, *N* = 3; mean ± s.e.m.; Fig. 6c). In order to explain this difference of the extent of cellular itineraries, the durations of migratory activity were evaluated for both genotypes. It emerged that the Tnc-deficient neuroblasts were motile for a mean time sequence of 5088 ± 124 min (*n* = 32, *N* = 3), whereas wild-type neuroblasts moved for a mean of 3530 ± 206 min (*n* = 34, *N* = 3, *p* < 0.001; Fig. 6d) during the observation period. It appears that the wild type cells paused significantly more often than the Tnc-deficient neuroblasts, which may reveal an anti-migratory effect of the glycoprotein on cell motility. While this functional trait of the generated neuroblasts was altered between the two genotypes, differentiation assays did not indicate a notable impact on cell fate (supplemental Fig. S3).

### Comparison of signaling pathways in the SEZ of Tnc-knockout mice in vivo

**Fig. 7:**
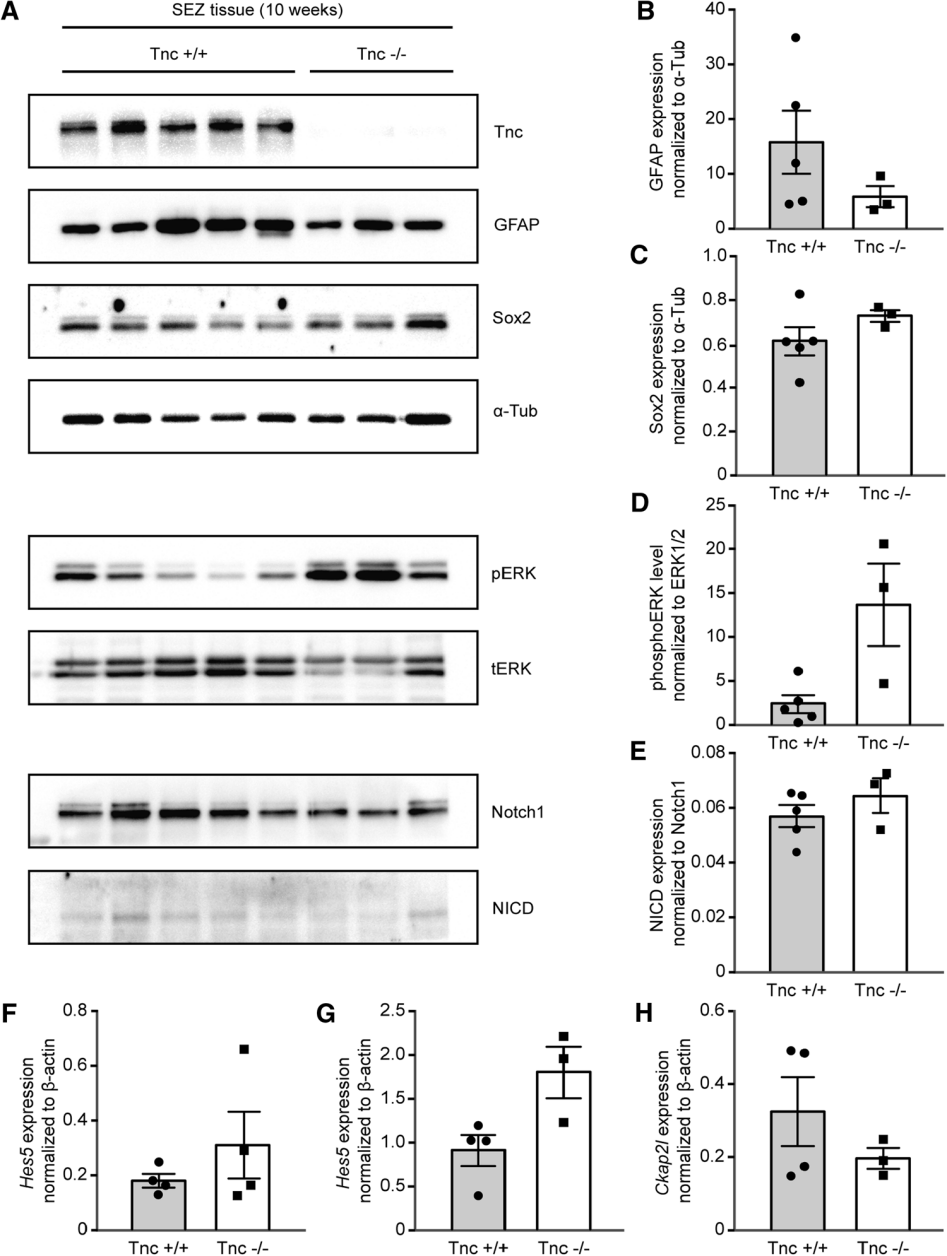
Analyses of gene expression in the lateral ventricular wall tissue from 10-week old mice. (A) Investigation of protein levels by Western blot technique. The analysis comprised five wild type samples (*N* = 5) and three Tnc-deficient samples (*N* = 3), as confirmed by anti-Tnc staining. The levels of GFAP and Sox2 in each sample were normalized to α-tubulin. **b** Quantification of GFAP and **c** of Sox2 signals displayed no statistically significant changes in Tnc-deficient SEZ tissue of animals. **d**, **e** An analysis of signaling pathways in the lateral ventricular wall tissue from 10-week old mice was carried out. Quantification of pERK signals demonstrated phosphorylation levels in Tnc-deficient SEZ tissue (**d**). Notch intracellular domain (NICD) expression in each sample was related to the corresponding Notch1 level (wild type *N* = 5, knockout *N* = 3). The ratio reflected an unaltered Notch pathway in knockout tissue (**e**). **f** Analysis of mRNA levels by RT-PCR suggested an implication of the Tnc-knockout in the Notch pathway activation and cell cycle progression. Tnc-deficient mice at 10 weeks of age expressed the target gene Hes5 in tissue isolated from the lateral ventricular wall (*N* = 4). **g** mRNA samples isolated from E15 neurospheres after 7 days in vitro confirmed Hes5 expression in Tnc-deficient cells (*N* = 4). **h** E15 neurospheres after 7 days of cultivation in suspension were tested for differences in the expression level of the cytoskeleton associated protein 2-like (Ckap2l) that encodes for a mitotic spindle protein. Ckap2l expression in Tnc-deficient neurospheres was unchanged compared to wild type neurospheres (*N* = 4). The resulting values showed considerable scatter resulting in large variations that precluded the detection of statistically significant differences. Data show mean ± s.e.m

It was known from previous studies that Tnc intervenes in critical signaling pathways [21]. Therefore, protein samples collected from the lateral ventricular wall of 10-week old wild type and knockout mice were analyzed by Western blotting (Fig. 7a). The astrocyte marker GFAP that is strongly expressed in type B stem cells [6] was not significantly decreased in Tnc-knockout samples (wild type: 15.8 ± 5.8 AU, *n* = 5, *N* = 5; Tnc knockout: 5.8 ± 1.9 AU, *n* = 3, *N* = 3, *p* = 0.14; Fig. 7b). The stem cell marker Sox2 varied barely between wild type and knockout (wild type: 0.61 ± 0.06 AU, *n* = 5, *N* = 5; knockout: 0.73 ± 0.03 AU, *n* = 3, *N* = 3; Fig. 7c). In addition to this result, immunohistochemical stainings for GFAP and Sox2 were performed (Supplemental Fig. S4). This analysis could further support the observation of the Western blots (see Fig. S3). Thus, the classical stem cell markers were negligibly affected by the depletion of Tnc from the aNSPC niche of the lateral ventricle. The same set of protein samples was also analyzed with emphasis on developmentally relevant signal transduction pathways. A particular attention was devoted to mechanisms that regulate cell cycle progression, as this process was modified by Tnc deficiency in the in vitro assays. The ERK signaling path was assessed by determining the level of phosphorylated ERK (pERK), as normalized to the corresponding total ERK1/2 (tERK) signal (wild type: 2.6 ± 1.0 AU, *n* = 5, *N* = 5; Tnc knockout: 13.8 ± 4.7 AU, *n* = 3, *N* = 3, *p* = 0.07 in Mann–Whitney *U* test; Fig. 7d). The seeming difference of activation in the knockout did not, however, achieve significance.

Furthermore, the Notch/delta pathway was examined that is involved in the maintenance of the aNSPC compartment [43]. The proportion of cleaved Notch1 receptor (Notch intracellular domain, NICD) serves as an indicator of the activation of this pathway and was normalized with regard to the total amount of Notch1. No modification could be found (wild type: 0.057 ± 0.004 AU, *n* = 5, *N* = 5; knockout: 0.065 ± 0.006 AU, *n* = 3, *N* = 3; Fig. 7e). The Notch/delta pathway controls downstream the transcription factor hairy/enhancer of split (Hes5) [44]. The message level of Hes5 was monitored by RT-PCR in tissue samples of Tnc-knockout mice at 10-weeks of age (wild type: 0.19 ± 0.03 AU, *n* = 4, *N* = 4; knockout: 0.32 ± 0.12 AU, *n* = 4, *N* = 4, *p* = 0.686; Fig. 7f). Neither could a significant difference be detected in cultures of cortical neurospheres derived from E15 mice (wild type: 0.91 ± 0.18 AU, *n* = 4, *N* = 4; knockout: 1.80 ± 0.25, *n* = 3, *N* = 3, *p* = 0.057; Fig. 7g).

As Tnc affects the duration of the cell cycle, genes involved in its regulation were of interest. In a previous study using gene arrays, the expression level of some cell cycle-related genes was found modified in the spinal cord of embryonic Tnc-deficient mice [24]. The cytoskeleton-associated protein 2-like protein (Ckap2l/Radmis) is required for mitotic spindle formation and cell cycle progression in neural progenitor cells [45]. We found no reduction of Ckap2l mRNA expression in cortical neurosphere cultures of E15 Tnc-knockout mice (wild type: 0.32 ± 0.09 AU, *n* = 4, *N* = 4; Tnc knockout: 0.19 ± 0.02 AU, *n* = 3, *N* = 3, *p* = 0.847; Fig. 7h′). Considering our results, we hypothesize that Tnc deficiency has an impact on signaling pathways that are relevant for the regulation of cell proliferation and cell cycle progression. The detailed mechanism and participating proteins remain to be investigated in the future, as the observed differences were not statistically significant. This may be a consequence of the fact that the NSPCs represent only a small fraction of the cells present in the tissue preparations used for analysis, while the pathways under consideration are shared by a large variety of cells [11]. Thus, background effects and the resulting variation of signal intensities may have obscured the NSPC-specific changes and precluded the obtention of statistical significance in our approach.

## Discussion

Quiescence and proliferation of adult neural stem cells are regulated by a complex interplay of extrinsic and intrinsic factors [46]. Our results show that the glycoprotein Tnc has a strong influence on the proliferation of aNSPCs obtained from the SEZ investigated by time-lapse video microscopy and lineage tracking in vitro. Adult neural stem cells originate from a subpopulation of slowly dividing neural progenitors during development, a process that requires the ongoing expression of the cell cycle inhibitor P57^Kip2^ [47]. In general, the cell cycle length of embryonic neural stem cells increases, owing to an expansion of the G1 phase [48, 49]. A link between cell cycle length and differentiation has been noted. For example, neural stem cells shorten the S-phase upon commitment to neuron production and the lengthening of the cycle can lead to premature neurogenesis [50, 51]. This may be linked to the reduced phosphorylation and activity of neurogenin 2 as a consequence of decreased CDK activity [52]. At any given time point about 10% of the neural stem cells proliferate in the adult niche (reviewed in [53]). Previous studies have distinguished resting and/or quiescent from activated stem cells. Explicit markers for quiescent stem cells have not yet been defined, although these cells seem to express higher levels of Sox9 and Id2 [13, 54]. Furthermore, transcriptome studies have revealed that quiescence is associated with the expression of genes related to cell–cell communication, cell adhesion and extracellular matrix [55].

Processes such as injury can lead to activation of neural stem cells that eventually express the EGF receptor (EGFR), a marker of the proliferating neural stem cells [56]. The cultivation of SEZ cultures in suspension revealed a reduced neurosphere formation capacity in Tnc-deficient cultures in response to EGF treatment. This could result from a decreased expression level of the EGFR. Interestingly, the transition towards the EGFR expressing NSCPs is retarded in the E12 telencephalon [28] and in the E15 spinal cord [24] of Tnc knockout mice. In adult mice, the presence of the EGFR discriminates between quiescent (EGFR-negative) and active (EGFR-positive) aNSPCs [57, 58]. Interestingly, Tnc activates the EGFR via its egf-type repeats, and the deletion of the gylcoprotein might thus disrupt a potential autokrine loop in aNSPCs [59, 60]. This loop is presumably relevant in our video recording experiments, where EGF was ommited from the medium. Thus, a reduced responsiveness to EGF might indicate a shifted proportion between two subpopulations of aNSPCs and does not necessarily point towards a generally reduced stem cell pool in Tnc-deficient mice. This explanation is in agreement with an in vivo approach of stem cell quantification in the adult Tnc-knockout niche using BrdU labeling, where no striking changes were detected in comparison to *Tnc*^±^ heterozygous mice [40]. According to this view, Tnc would somehow intervene in the transition between quiescence towards activation of the adult neural stem cells. Interestingly, the upregulation of the EGFR is promoted by the cytokine FGF2 [41, 61]. When aNSPCs were cultivated in a medium containing both EGF and FGF2, the latter cytokine may have overriden the delay of EGF-responsiveness in *Tnc*^*−/−*^ cells, as no reduction of neurospheres had been observed under these conditions [40]. Upregulation of the EGFR is an indicator, but not the sole feature of activated neural stem cells that are also associated with elevated levels of *Egr1*, *Fos*, *Sox4*, *Sox11* and downstream regulated genes [13]. The resulting shift of transcriptomes allows for a clear-cut separation of quiescent versus activated neural stem cells on the base of principal component analysis [62].

Activated adult proliferating neural stem cells behave similarly as their embryonic counterparts and their continuous production would lead to the exhaustion of the aNSCP pool [62]. Therefore, the maintenance of the quiescent population is of high biological significance. The question is of interest how ECM compounds can modulate the activation, the proliferation and the cell cycle length of neural stem cells [63]. Quiescent neural stem cells reside primarily in the G0, and some on the G2 phase of the cycle (reviewed in [53]). Upon activation, cell cycle promoting genes such as Cdk1 and Mki67 become upregulated [64], and the duration of the cycle is mainly determined by the G1 phase. The persistence of stem cell quiescence is trongly supported by integrins and the integrin-linked kinase ILK, underlining an important role of the ECM [63, 65]. Interestingly, Tnc interacts with several integrin ligands [66]. How the hypothesized gate keeper function of Tnc might operate is, however, currently unkown.

A salient finding of our analysis was the reduced length of the cell cycle in the Tnc knockout aNSCPs on PDL and LN1 substrates. This was reminsicent of a shorter cell cycle determined by time lapse video microscopy in E15 spinal cord NSPCs [29]. These observations are in agreement with cell proliferation studies in Tnc knockout mice in vivo. Indeed, increased mitotic activity has been reported in the E12 telencephalon, the E15 spinal cord, the early postnatal forebrain and the embryonic retina [20] of Tnc knockout mice [24, 27, 28]. Thus, a reduced exit of aNSPCs from quiescence to the activated state might be compensated by an increased proliferation of transient amplifying progenitors or neuroblasts. Consistent with this assumption the number of neuroblast clusters along the lateral ventricle and the number of OPCs in the optic nerve were significantly increased in *Tnc*^*−/−*^ mice [40]. With regard to further downstream mechanisms related to cell cycle control, the cytoskeleton-associated protein 2-like protein (Ckap2l/Radmis) was tested in *Tnc*^*−/−*^ SEZ knockout tissue, without, however, revealing significant differences. A reduction had previously been described in embryonic spinal cord based on transcriptome analysis [24].

The influence of Tnc on cell proliferation is controversially being discussed, as Tnc elimination caused opposite results, depending on the experimental context and cell type [67, 68]. The cultivation on a Tnc substrate accelerated the cell cycle in the majority of proliferating wild-type aNSPCs but elongated it in the subset of slow-dividing wild-type aNSPCs_late_. This subpopulation behaved as expected for genuine aNSPCs, distinct from fate-restricted progenitors [8]. Both proliferating populations may be considered as progenitors isolated at different stages of their respective lineage progression [9]. The opposite response to the ECM component Tnc might indicate differences in the receptor expression that eventually impact signaling mechanisms to alter cell cycle progression. This phenomenon was not noted in Tnc-knockout aNSPC_late_ cultures where a compensation caused by coated Tnc failed. It seems that the absence of Tnc in the adult neural stem cell niche of knockout mice desensitized the *Tnc*^*−/−*^ aNSPC_late_ for this compound. Another possible explanation would be a threshold effect, where the coating protein on the culture surface has to be supplemented by cell secreted Tnc protein to reach an effective concentration. It remains still elusive whether Tnc expression in the SEZ of adult mice has the function of maintaining the stem cell pool. If this were the case this would favor a faster depletion of the stem cell pool in Tnc-deficient populations. This interpretation remains to be tested in a future study with a larger sample of SEZ cultures and a broad statistical basis.

It has been described for several cell types that LN1 promotes cell proliferation and neurogenesis [34–36, 69]. The accelerating effect on cell cycle progression was also observed in our experiments for the mitotic population of the primary SEZ cultures from wild type and Tnc-knockout mice. For both ECM compounds of interest investigated in this study, integrins represent important receptors [70, 71]. Thus, it is conceivable that the regulatory effects enacted by these substrate proteins are mediated by integrins [72, 73]. This most probably involves different heterodimeric receptors, as the reaction of slow-dividing aNSPCs_late_ to the LN1 and Tnc substrates was not congruent. In support of this assumption, a distinct expression pattern of integrin variants during lineage progression has been reported [63].

Several reports suggest that Tnc has an impact on migration [74–76]. In particular in pathologic contexts such as cancer, a promoting effect on migration and metastasis has been attributed to Tnc [77, 78], which might be mediated via integrins [79, 80]. Our in vitro experiments with aNSPCs from the *Tnc*^*−/−*^ deficient SEZ revealed increased migration trajectories of neuroblasts that were caused by elongation of movement episodes. This is reminiscent of increased migration of oligodendrocyte precursors observed in Tnc-deficient rats [27]. Tnc expression was found in the pathway of the rostral migratory stream [40, 81] where neuroblasts migrate along to colonize the olfactory bulb and integrate into local neuronal networks [82]. The Tnc glycoprotein might have a guidance function there as it serves as inhibitory boundary molecule for olfactory sensory neurons [83]. Along these lines, early studies on Tnc in cell cultures characterized the glycoprotein as anti-adhesive [38, 84]. Anti-adhesion was also seen in the videos generated in this study with respect to aNSPCs, although not as pronounced as for embryonic neural progenitors (unpublished observations). Changes of the intensity of substrate adhesion might be an indirect cause of the observed results and has been discussed as a potential mechanism to influence migration and proliferation [85].

We examined the GFAP protein level in SEZ samples with regard to potential alterations that could point to structural changes in the niche of Tnc-knockout mice. GFAP is a marker that is expressed by niche astrocytes and aNSPCs [40]. We observed variable expression levels in wild type and knockout tissues that did not allow for statistically stringent conclusions. Minor structural changes had already been described by previous studies of the SEZ niche in *Tnc*^*−/−*^ knockout animals [40]. The latter study for example described an increased number of neuroblast clusters around the lateral ventricle of *Tnc*^*−/−*^ null mice. This is in agreement with our hypothesis of a higher rate of neuroblast generation by *Tnc*^*−/−*^ aNSPCs in vitro.

To gain mechanistic insight, we investigated a potential intervention of Tnc in signaling pathways in the adult SEZ. The ERK pathway activity is of interest, because this pathway is linked to the regulation of migration in numerous contexts [86], which may mechanistically explain the enhanced migration of knockout cells observed here. The increased rate of phosphorylated ERK could also be the cause for faster cell cycling as this pathway is known to regulate cell cycle progression and transition from G1 to S-phase [87, 88]. Activation of the ERK1/2 pathway in primary embryonic cortical neurons by the synthetic biomimetic VSWRAPTA peptide derived from the primary Tnc sequence has been reported [32]. However, we did not obtain conclusive evidence for alteration of the pathway upon Tnc knockout. It has to be kept in mind that the NSPCs represent a small population whereas the ERK1/2-pathway is shared by numerous CNS cell types. A large scatter of ERK-activation values presumably resulting from the variability of sample preparations ensued that may have obscured the statistical significance. Taken together, the implication of the ERK1/2 pathway in mediating reactions to Tnc in the niche environment remains to be evaluated.

## Conclusions

Taken together, our data provide novel insights into the role of Tnc in the stem cell niche of the adult SEZ. First, in the adult as well as in the embryonic CNS elimination of Tnc leads to a delayed activation of stem cells, as reflected by a tampered response towards the cytokine EGF. Whether Tnc solely modulates the timing or affects stem cell activation by another mechanism remains to be established. Second, Tnc exerts a regulatory role in cell cycle progression of aNSPCs. Thereby, Tnc intervenes in cell cycle progression and its elimination consequently reduces cell cycle length. This holds true for both aNSPCs and embryonic neural stem cells, as shown in earlier studies. Defined ECM components such as Tnc and LN1, both parts of the adult stem cell niche, influence the proliferation dynamics of the dividing aNSPC population. Third, a closer inspection revealed different effects depending on lineage progression of aNSPCs, with particular features of the slowly dividing aNSPCs_late_ subpopulation that closely resembles type B stem cells of the niche. The impact of the ECM most probably is mediated by alterations in key signaling pathways of cell cycle regulation in the Tnc-knockout mice in vivo. Due to technical limitations of our approach this concept has to be verified in future studies. Fourth, Tnc ablation did not modify the differentation of aNSPCs into distinct lineages. However, Tnc shortened the migration trajectories of neuroblasts, the major offspring of aNSPCs, by reducing the duration of migratory episodes.

## Supplementary Information

Below is the link to the electronic supplementary material.

Supplementary file1 (DOCX 3340 KB)

Supplementary file2 (AVI 9215 KB)

Supplementary file3 (AVI 8648 KB)

Supplementary file4 (AVI 8193 KB)

Supplementary file5 (AVI 9570 KB)

## Data Availability

The materials used in this study are available to any qualified researcher upon reasonable request addressed to A.F.
