# Supplementary material for: The extracellular matrix molecule tenascin-C modulates cell cycle progression and motility of adult neural stem/progenitor cells from the subependymal zone
Source: Cell Mol Life Sci. 2022 Apr 16;79(5):244. doi: 10.1007/s00018-022-04259-5 (PMC9013340; doi:10.1007/s00018-022-04259-5)
Supplement: Supplementary file 1 — Supplementary file1 (DOCX 3340 KB) [file 18_2022_4259_MOESM1_ESM.docx]

**The extracellular matrix molecule tenascin-C modulates cell cycle progression and motility of adult neural stem cells from the subependymal zone**

**Supplemental data**

**
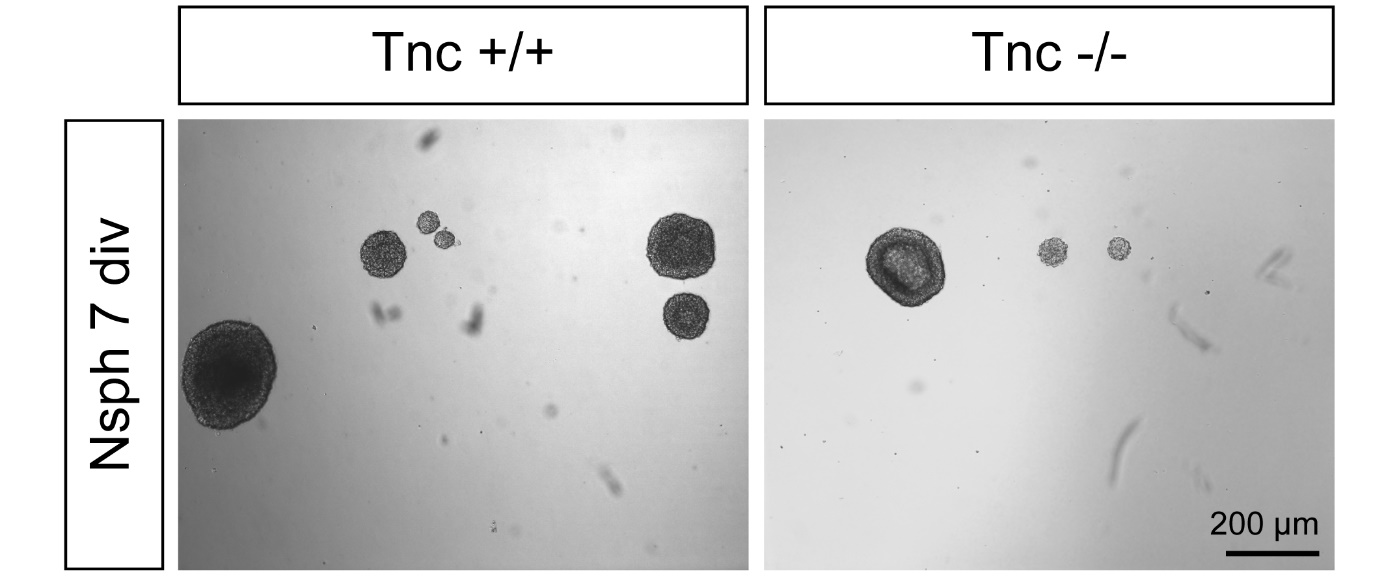
**

**Figure S1.** Neurosphere assay. Adult SEZ-derived cell suspensions obtained from 11-weeks old mice were cultivated in the presence of 20 ng/ml EGF at a density of 1000 cells/ml. The percentage of plated cells generating neurospheres after 7 days in vitro is significantly reduced in Tnc-knockout mice (N=5).

**
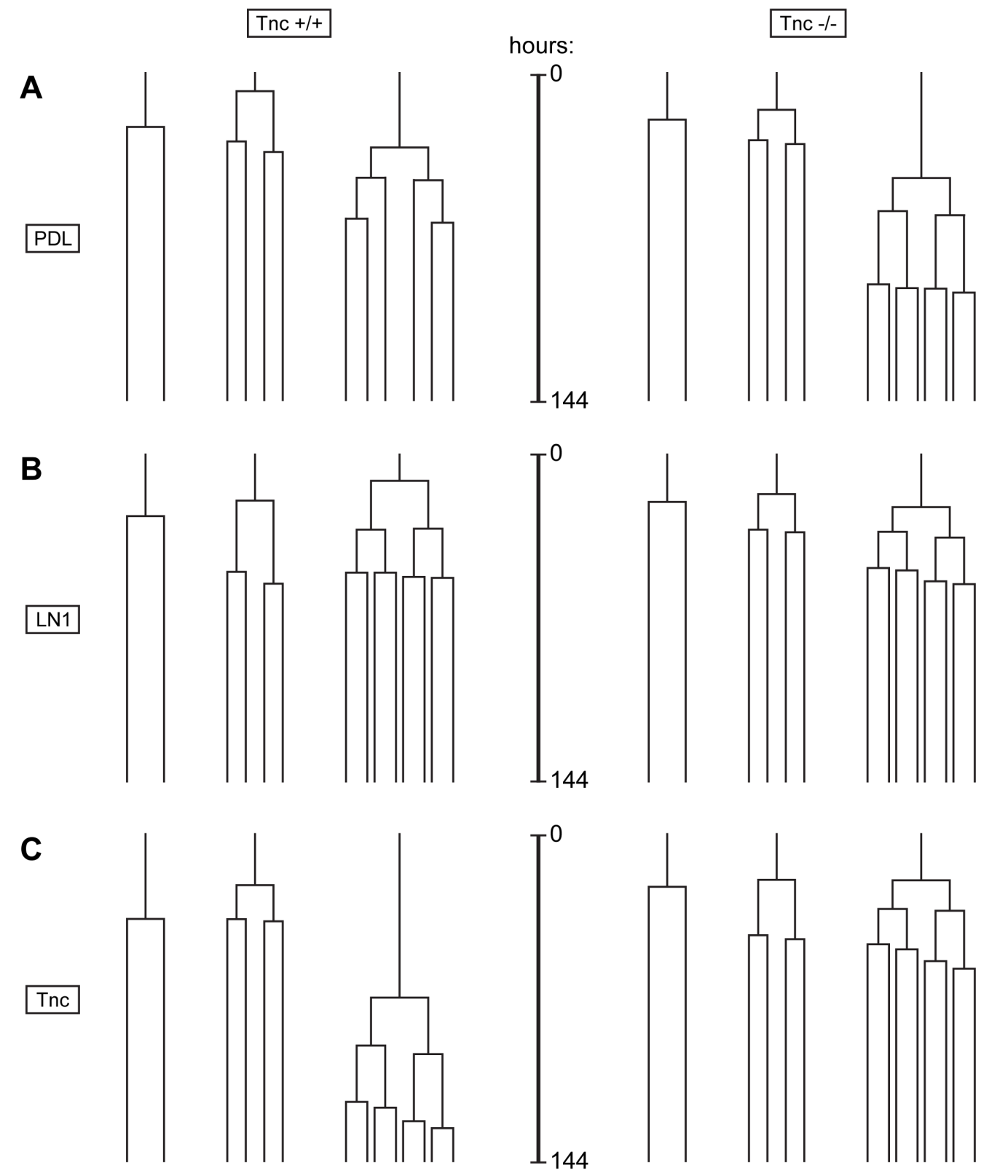
**

**Figure S2.** Diversity of lineage trees. This figure shows more examples of lineage trees for wildtype and Tnc knockout cells to strengthen how diverse the proliferative population acts within one condition. (A) PDL control condition, (B) cultivation on LN1 substrate and (C) cultivation on Tnc substrate. Approximately 50% of the pedigrees showed only one cell division (here always shown on the left side of each group). One third divided twice within the observation period (examples shown here in the middle of each group) and only 10-15% showed more complex lineage trees like those displayed on the right side of each group.

**
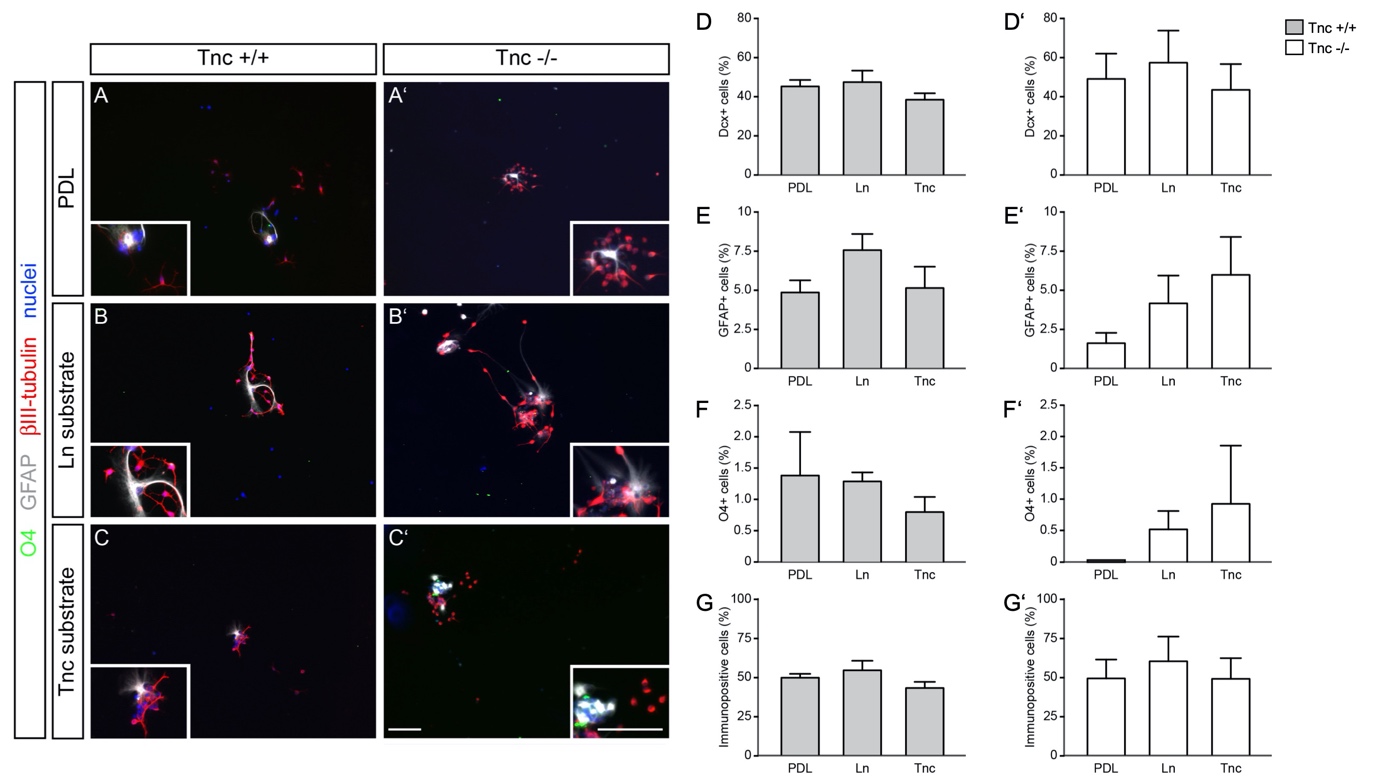
**

**Figure S3.** Differentiation assay of wildtype and Tnc-deficient aNSPCs cultures shows no alterations in cell fate. (A) Cells isolated from the lateral ventricular wall of 9 to 11-week old mice were prepared according to a protocol derived from published procedures [1], plated onto different substrates and cultivated for 7 days in defined medium devoid of cytokines. Immunofluorescence staining was performed to analyze the proportions of doublecortin- (Dcx+), glial fibrillary acidic protein- (GFAP+) and sulfatide-positive (O4+) cells in cultures of both *Tnc^+/+^* and *Tnc^-/-^* genotypes. Scale bar: 200 µm. (D-G) Quantification of the cell types after 7 days *in vitro* indicates only minor effects of extracellular matrix substrates on aNSPC differentiation (N=5; PDL: n=2831, LN1: n=3563, Tnc: n=2027). (D, D’) The percentages of Dcx+ cells in wildtype and Tnc-knockout cultures from the SEZ did not differ between the three substrate conditions. Dcx+ neuroblasts represented the major cell type in these cultures after 7 days of differentiation. (E, E’) Evaluation of the astrocyte fraction in wildtype cultures using GFAP staining disclosed no significant changes. (F, F’) O4+ cells were rare after 7 days of differentiation, but modified neither by Tnc-depletion in the knockout, nor by distinct ECM substrates. (G, G’) Displays the fraction of cells immunopositive for any of the markers, that comprised nearly half of the cells examined. This proportion did not vary between the substrates tested. Data show mean ± s.e.m. Kruskal-Wallis test with Dunn’s multiple comparison were performed for statistical analysis.

**
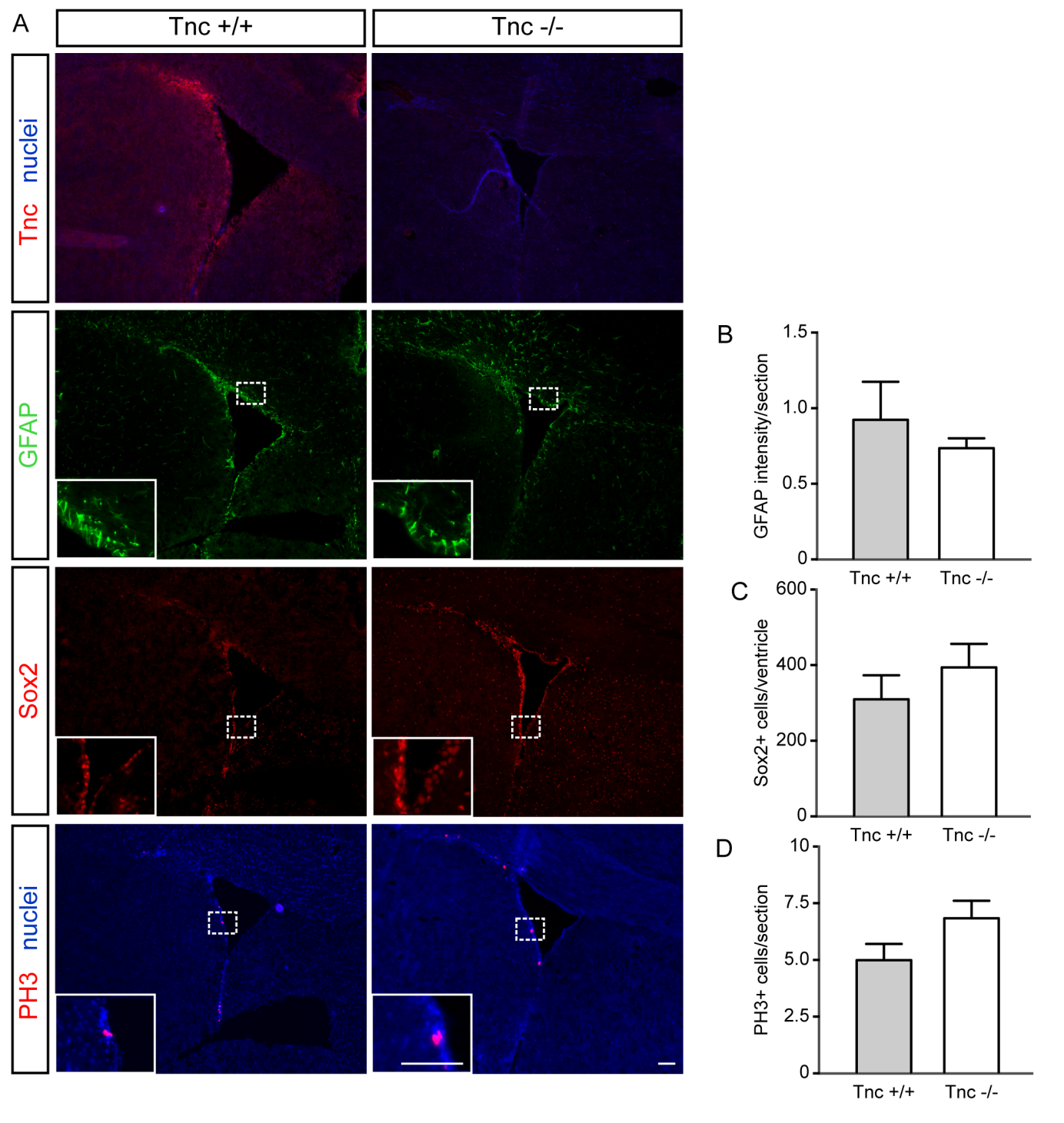
**

**Figure S4.** Analysis of the adult SEZ in vivo by immunohistochemistry. (A) Immunohistochemistry of frontal sections from 11-week-old wildtype (left) and Tnc knockout mice (right) was performed. Measurements of the staining intensity showed a slightly reduced GFAP signal in knockout sections compared to wildtype sections (B). The number of Sox2-immunopositive cells surrounding the lateral ventricle was slightly increased in the knockout brains (C), which somehow resembled the results from the protein analysis. Nevertheless, in both methods, there were no hints that these two markers or cell populations seemed to be significantly altered when Tnc is missing. Furthermore, we determined the amount of phospho-histone h3 (PH3)-positive cells in both genotypes (D). Generally, there was a very limited number of cells which expressed the corresponding antigen. PH3 is only expressed in cells undergoing mitosis. Thus, we could not find significant changes between the two groups.

**Table S1. Cell cycle lengths of the proliferating population within the SEZ cultures in hours *in vitro*.**

| Genotype | Substrate | Gene-ration | Median | Mean | Upper quartile | lower quartile | n |
| --- | --- | --- | --- | --- | --- | --- | --- |
| Tnc +/+ | PDL | 1st | 9,67 | 12,93 | 18,25 | 5,75 | 171 |
|  |  | 2nd | 21,83 | 23,82 | 27,63 | 17,92 | 157 |
|  |  | 3rd | 17,17 | 19,12 | 19,80 | 15,25 | 81 |
|  |  | 4th | 25,33 | 22,67 | 27,38 | 16,67 | 21 |
|  | Laminin | 1st | 9,50 | 12,77 | 19,58 | 4,71 | 185 |
|  |  | 2nd | 20,97 | 22,32 | 25,60 | 16,58 | 176 |
|  |  | 3rd | 16,75 | 18,25 | 20,67 | 15,08 | 125 |
|  |  | 4th | 17,00 | 18,13 | 20,08 | 15,25 | 57 |
|  | Tenascin-C | 1st | 10,83 | 13,82 | 20,33 | 4,38 | 141 |
|  |  | 2nd | 19,33 | 20,97 | 24,47 | 16,00 | 145 |
|  |  | 3rd | 16,58 | 17,85 | 19,67 | 15,08 | 67 |
|  |  | 4th | 16,25 | 18,32 | 22,50 | 15,67 | 7 |
| Tnc -/- | PDL | 1st | 11,33 | 13,63 | 19,30 | 6,07 | 150 |
|  |  | 2nd | 18,67 | 20,20 | 24,05 | 14,88 | 117 |
|  |  | 3rd | 17,00 | 19,37 | 22,73 | 14,75 | 106 |
|  |  | 4th | 18,25 | 19,00 | 21,93 | 14,75 | 54 |
|  | Laminin | 1st | 12,17 | 14,33 | 20,58 | 5,08 | 107 |
|  |  | 2nd | 18,13 | 19,88 | 22,92 | 14,75 | 106 |
|  |  | 3rd | 16,25 | 16,70 | 18,42 | 13,67 | 79 |
|  |  | 4th | 15,00 | 15,45 | 16,55 | 13,63 | 29 |
|  | Tenascin-C | 1st | 10,92 | 13,48 | 18,75 | 5,33 | 93 |
|  |  | 2nd | 17,58 | 19,78 | 22,92 | 14,25 | 103 |
|  |  | 3rd | 16,13 | 17,17 | 19,38 | 13,77 | 96 |
|  |  | 4th | 15,75 | 18,00 | 17,25 | 13,92 | 52 |

**Table S2. Cell cycle lengths of the slow-dividing aNSPC_late_ subpopulation in the SEZ cultures in hours *in vitro*.**

| Genotype | Substrate | Gene-ration | Median | Mean | Upper quartile | lower quartile | n |
| --- | --- | --- | --- | --- | --- | --- | --- |
| Tnc +/+ | PDL | 1st | 98,92 | 91,12 | 112,67 | 57,97 | 13 |
|  |  | 2nd | 16,80 | 17,33 | 20,00 | 14,54 | 10 |
|  |  | 3rd | 17,17 | 20,73 | 24,80 | 15,92 | 9 |
|  |  | 4th | 18,79 | 18,79 | 20,00 | 17,58 | 2 |
|  | Laminin | 1st | 73,58 | 80,97 | 100,73 | 68,00 | 18 |
|  |  | 2nd | 17,50 | 18,55 | 21,32 | 16,00 | 24 |
|  |  | 3rd | 16,46 | 16,68 | 18,83 | 14,96 | 32 |
|  |  | 4th | 16,33 | 16,77 | 19,54 | 14,32 | 22 |
|  | Tenascin-C | 1st | 70,25 | 75,35 | 86,92 | 62,50 | 11 |
|  |  | 2nd | 19,17 | 19,18 | 21,67 | 15,92 | 27 |
|  |  | 3rd | 18,83 | 20,03 | 22,40 | 16,35 | 24 |
|  |  | 4th | 23,25 | 23,25 | 24,00 | 22,50 | 4 |
| Tnc -/- | PDL | 1st | 78,92 | 82,17 | 90,02 | 72,83 | 12 |
|  |  | 2nd | 13,92 | 14,41 | 15,50 | 12,46 | 20 |
|  |  | 3rd | 15,92 | 17,27 | 18,58 | 14,08 | 37 |
|  |  | 4th | 15,96 | 17,67 | 19,77 | 14,40 | 36 |
|  | Laminin | 1st | 70,92 | 80,23 | 95,80 | 61,38 | 9 |
|  |  | 2nd | 14,04 | 14,63 | 17,08 | 12,25 | 14 |
|  |  | 3rd | 15,29 | 15,05 | 17,67 | 12,35 | 26 |
|  |  | 4th | 15,67 | 16,33 | 17,88 | 14,90 | 18 |
|  | Tenascin-C | 1st | 95,75 | 101,25 | 120,58 | 85,38 | 9 |
|  |  | 2nd | 15,17 | 15,35 | 17,18 | 13,67 | 16 |
|  |  | 3rd | 14,67 | 14,88 | 15,75 | 13,50 | 23 |
|  |  | 4th | 15,96 | 15,98 | 17,30 | 14,68 | 22 |

**Table S3. List of primers used for RT-PCR**

| Primer | Sequence | Annealing Temp. | Accession  No. | Product size (bp) |
| --- | --- | --- | --- | --- |
| β-actin forw.  β-actin rev. | TATGCCAACACAGTGCTGTCTGG  AGAAGCACTTGCGGTGCACGATG | 60°C | NM_007393.5 | 247 |
| Ckap2l forw.  Ckap2l rev. | TGCGTTCTGCAAAAATCAAG  TGAGTCCTGGGAGCAGTCTT | 58°C | NM_181589.3 | 218 |
| EGFR forw.  EGFR rev. | ACCCAACTGGGCACTTTTGA  TGTCACCACGTAGTTTCGGG | 58°C | NM_207655.2 | 792 |
| Hes5 forw.  Hes5 rev. | AAGTACCGTGGCGGTGGAGAT  CGCTGGAAGTGGTAAAGCAGCTT | 60°C | NM_010419.4 | 354 |

**Table S4. List of primary antibodies:**

| Antibody | Producer | Cat. No. | RRID/Reference |
| --- | --- | --- | --- |
| Mouse anti-a-tubulin | Sigma | T6199 | AB_477583 |
| Mouse anti-GFAP | Sigma | G3893 | AB_477010 |
| Rabbit anti-Sox2 | Millipore | AB5603 | AB_2286686 |
| Rabbit anti-Notch1 | Cell Signaling | 3608S | AB_2153354 |
| Rabbit anti-cleaved Notch1 | Cell Signaling | 4147S | AB_2153348 |
| Rabbit anti-ERK | Santa Cruz | sc-94 | AB_2140110 |
| Rabbit anti-pERK | Santa Cruz | sc-16982 | AB_2139990 |
| Rabbit anti-EGFR | Santa Cruz | sc-03 | AB_631420 |
| Rabbit anti-Tnc  Rabbit anti-Tnc | Batch KAF14.1  Batch KAF12 |  | Both [2] |
| Mouse anti-O4 | Millipore | MAB-345 | AB_94872 |
| Mouse anti-bIII tubulin | Sigma | T8660 | AB_477590 |

**Supplemental movie 1 (M1).** Adult wild-type neural stem cells cultivated on poly-D-lysine. aNSPCs were plated on poly-D-lysine and cultivated for six days. A clone of dividing cells forms a small sphere, indicating that cell adhesion is stronger than cell-substrate attachment (left lower quadrant). A comparable behavior was seen on substrates coated with LN1 or Tnc (not shown).

**Supplemental movie 2 (M2).** Adult *Tnc^-/-^* neural stem cells cultivated on poly-D-lysine. *Tnc^-/-^* aNSPCs were plated on poly-D-lysine and cultivated for six days. A clone of dividing cells displays three division events, resulting in 8 cells (right lower quadrant). Tnc-deficient cells displayed a better substrate attachment, presumably reflecting the absence of the anti-adhesive ECM compound [2].

**Supplemental movie 3 (M3).** Adult *Tnc^-/-^* neural stem cells cultivated on LN1. *Tnc^-/-^* aNSPCs were plated on LN1 and cultivated for six days. A clone of dividing cells displays three division events, resulting in 8 cells (right upper quadrant). Tnc-deficient cells on LN1 extended considerable processes in response to LN1. This was not the case with Tnc-secreting wild-type cells.

**Supplemental movie 4 (M4).** Adult *Tnc^-/-^* neural stem cells cultivated on Tnc. *Tnc^-/-^* aNSPCs were plated on Tnc and cultivated for six days. A clone of dividing cells displays division events (right upper quadrant). Tnc-deficient cells on Tnc extended actively searching filopodia that, however, did not translate into stable processes, different from the situation on LN1. This presumably is a consequence of the anti-adhesive Tnc-rich substrate [2,3].

**References**

1. Ortega F, Costa MR, Simon-Ebert T, Schroeder T, Gotz M, Berninger B (2011) Using an adherent cell culture of the mouse subependymal zone to study the behavior of adult neural stem cells on a single-cell level. Nat Protoc 6 (12):1847-1859. doi:10.1038/nprot.2011.404

2. Faissner A, Kruse J (1990) J1/tenascin is a repulsive substrate for central nervous system neurons. Neuron 5 (5):627-637

3. May M, Denecke B, Schroeder T, Gotz M, Faissner A (2018) Cell tracking in vitro reveals that the extracellular matrix glycoprotein Tenascin-C modulates cell cycle length and differentiation in neural stem/progenitor cells of the developing mouse spinal cord. Biol Open 7 (7). doi:10.1242/bio.027730
